# Emulsions Using a Vortex-Based Cavitation Device: Influence of Number of Passes, Pressure Drop, and Device Scale on Droplet Size Distributions

**DOI:** 10.1021/acs.iecr.2c03714

**Published:** 2022-12-19

**Authors:** Abhijeet H. Thaker, Vivek V. Ranade

**Affiliations:** Multiphase Reactors and Intensification Group Bernal Institute, University of Limerick, LimerickV94T9PX, Ireland

## Abstract

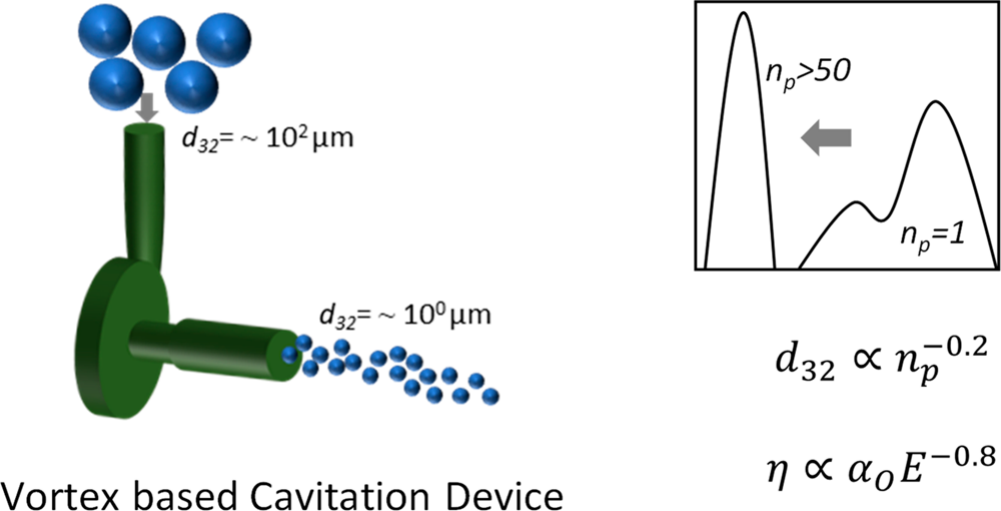

Liquid–liquid emulsions are used in a variety of industry sectors, including personal care, home care, food, and nutrition. The development of compact and modular systems and devices for creating emulsions with desired droplet size distribution (DSD) is becoming increasingly important. In this work, we have shown use of vortex-based cavitation devices for producing emulsions at nominal flow rate of 1 LPM and 20 LPM. We present new experimental results providing quantitative information on influence of multiple passes through the vortex based hydrodynamic cavitation (HC) device, type of oil and device scale on the breakage process and resulting DSDs. Multiple pass experiments were performed for generating oil-in-water emulsions containing 5 and 15% of oil. Rapeseed oil (RO) and tetrachloroethylene (TCE) were used as oil phases with densities of 915 and 1620 kg/m^3^, respectively. The effect of pressure drop across the HC device in the range of 50–250 kPa on DSD was examined. The HC device was shown to exhibit significant higher efficiency compared to alternative emulsion making devices (i.e., homogenizers, venturi, and orifice-based HC devices), and the Sauter mean drop size was found to reduce from 66 μm to less than 2 μm after about 50 passes in all the device scales. The DSD of the RO–water system showed a bimodal nature, whereas monomodal DSD was found for TCE–water system. Preliminary simulations using the computational fluid dynamics–population balance model (CFD-PBM) models developed in the previous work indicated the inadequacy of developed models to capture the influence of cavitation on DSDs. By carrying out Hinze scale analysis of bimodal DSD, we for the first time showed the existence of two different mechanisms (one based on conventional turbulent shear and the other based on collapsing cavities) of droplet breakage in HC devices. The order of magnitude of turbulence energy dissipation rates generated due to collapsing cavity estimated using Hinze scale analysis showed good agreement with the values reported from cavity dynamics models. The presented experimental results and analysis will be useful for researchers and engineers interested in developing computational models and compact devices for producing emulsions of the desired DSD.

## 1 Introduction

Several commercial products in personal care (cosmetics, beauty care), home care (paints, room fresheners), and food (milk products, ice creams) industries are formulated using liquid–liquid emulsions. One of the most important critical quality attributes (CQA) in emulsions is droplet size distribution (DSD), which has an impact on other qualities including rheology, appearance, stability, and so on. Therefore, a lot of work has been done and is being done to develop methods for producing emulsions with desired DSD. Based on the product applications, emulsions are classified into three categories such as macroemulsions (drop size: 1–100 μm), nanoemulsions (drop size: 20–500 nm), and microemulsions (drop size: 10–100 nm).^1^ A variety of emulsion preparation techniques and equipment are available, including impeller agitated vessels,^2−4^ rotor-stators,^1,5,6^ high-pressure homogenizers,^6,7^ colloid mills,^7,8^ ultrasonication,^9,10^ hydrodynamic cavitation,^11−17^ membranes and microchannels,^18^ and so on. The ultrasonic systems and membranes are heavily utilized in laboratory-scale or developmental systems. The major drawbacks of these equipment systems are difficulties in scale-up and higher energy requirements. The agitated vessels, high-pressure homogenizers, or rotor-stators and hydrodynamic cavitation (HC) are industrial production equipment.^18^

The emulsification process occurred in the agitated vessels due to the turbulence shear generated through different types of impellers.^2−4^ Groeneweg et al.^2^ used a stirred vessel to generate paraffin oil in water emulsion for different impeller speeds. They mentioned that the emulsion generated in the stirred vessel was due to the transitional flow in which the turbulence flow was generated near the impeller region. The flow might be laminar at larger distances from the impeller, resulting in wide drop size distributions. They also differentiate the viscosity-dominated and inertia-dominated droplet breakup zones based on the drop sizes.^2^ Khalil et al.^4^ investigated the drop breakup process using two different impeller types to generate emulsion, i.e., flat blade propeller and Rushton turbine. They found faster droplet breakage in the Rushton turbine because the Ruston turbine operates at a higher power number than the flat blade propeller, leading to high energy dissipation rates and rapid droplet breakage.^4^ Emulsions are formed in high-pressure homogenization or high-energy rotor-stator devices due to the extreme elongational, shear stress, and pressure differences that lead large drops to break into smaller droplets.^1,5,6^ These equipment systems usually circulate emulsions multiple times to obtain the desired mean drop size reduction. In colloidal mills, pre-emulsion is passed through the narrow gap between the rotor and stator. The rotor rotating at high angular velocity and drop tends to stretch due to the very high shear rate (10^4^–10^6^ 1/s) that leads to drop breakage.^7,8^ The major drawbacks of these equipment systems are high energy consumption and complex internal arrangements. Ultrasonication has recently been presented as an efficient emulsification technology.^9^ In the ultrasonication process, cavitation induced through acoustic methods and shock waves using ultrasonic sonotrodes results in drop breakage.^10^ The major difficulties of ultrasonication for emulsification process are scale-up and higher energy requirements which limit the use of ultrasound technology for commercial emulsification processes. Apart from the aforementioned equipment, emulsion production by HC offers an attractive alternative to high-energy equipment.

The HC is a process of vapor cavity generation, growth, and collapse^19,20^ that results in strong shear and concentrated high-velocity jets, which can be used for drop breakage and emulsion formation. The energy consumption of HC is an order of magnitude lower than ultrasonication and is more amenable for scale-up.^11,21^ Recently, single-step homogenization with a controlled cavitation approach patented by SOLDO cavitators^22^ was used for emulsification and gained significant attention in commercial-scale emulsion generation for the food industry. Based on the geometric structure, HC devices can be classified as linear flow devices (such as orifices or venturi) and swirling flow-based HC devices (vortex diodes). A few articles are available for generating emulsion using the HC approach.^11,12,14−17,23^ Parthasarathy et al.^11^ used liquid whistle hydrodynamic cavitation (LWHCR) to create a palm oil based oil/water (O/W) submicrometer emulsion. The LWHCR is a linear flow orifice-based HC device. They investigated the influence of operating and geometrical parameters such as inlet pressure and knifelike blade and found a minimum drop size of ∼0.5 μm with a droplet size distribution polydispersity index (PDI) of 0.5. Furthermore, a similar setup of the LWHCR was employed to emphasize the application and potential scope of HC in the pharmaceutical industry.^12^ Ramisetty et al.^14^ performed HC experiments using circle and slit venturi type devices to generate coconut oil based O/W emulsions. They analyzed the influence of operating parameters such as inlet pressure, number of passes, dispersed phase volume fraction, and concentration of surfactants on the drop size distribution. The mustard oil based emulsion was generated with HC treatment using different shape and size of the orifice plate by Carpenter et al.^16^ They found that a circular-shaped single-hole orifice plate (having a lower perimeter and higher flow area) performed better in terms of the smallest droplet size than that of the other devices considered in their study. Using a relatively straightforward configuration of HC valve, Zhang et al.^15^ created an O/W emulsion with soybean oil as the base (10 vol %) with a pressure drop larger than 800 kPa. Due to less energy utilization than rotor-stators or high-pressure homogenizers, the aforementioned HC devices (LWHCR, venturi and orifice, HC valve) are an attractive alternative for conventional emulsion generation equipment. However, the major limitation of linear flow HC devices in industrial-scale manufacturing is the need for high inlet pressures (∼10^3^ kPa), susceptibility to erosion (and subsequent loss of performance), and less control over the resulting DSD of emulsions.^24^ Unlike these, vortex-based HC devices exhibit early inception of cavitation, and therefore, the inlet pressures required to generate emulsion is lower (∼10^2^ kPa) than the linear flow HC devices.^25^

The strongly swirling flow retains a cavitating core at the center and shields the cavitation device from erosion. Recently, we have investigated the single droplet breakage process in vortex-based HC device^26^ followed by the influence of oil volume fraction (over the range of 1–20%) on DSD of emulsions produced in a single pass through vortex-based HC device.^25^ The drop breakage and generated emulsion through single-pass HC treatment was systematically studied. Overall, a proof of concept for employing these vortex-based HC devices for emulsions^25,26^ was developed. We used focused beam reflectance measurement (FBRM) for determining the drop size distribution.^25^ Because of its inherent limitation, FBRM cannot measure droplet sizes smaller than 1 μm. When we repeated the measurements of DSD of emulsions produced in vortex-based devices using the MasterSizer (MS), we could observe that a significant number of droplets were smaller than 1 μm which were missed in our earlier FBRM analysis (see section 2 on experiments for more details on this). Therefore, in this work, we used the MS to quantify the full range of DSD emulsions produced using vortex-based HC devices.

In this work, we investigated the influence of multiple passes, pressure drop across HC device, device scale, and type of oil on the resulting DSD of emulsions. The computational fluid dynamics–population balance model (CFD-PBM) simulations were performed to predict drop size distribution of multiple passes. An attempt is made to analyze observed DSD and relate the observations to possible breakage pathways. The Hinze scale analysis was performed using the measured DSD and the values of turbulence energy dissipation rates generated through collapsing cavities were estimated and compared with the literature.^27^ The present work will be useful for improving computational models and for harnessing vortex-based devices for producing industrially relevant emulsions on scale.

## 2 Experiments

### 2.1 Experimental Setup and Procedure

**Figure 1:**
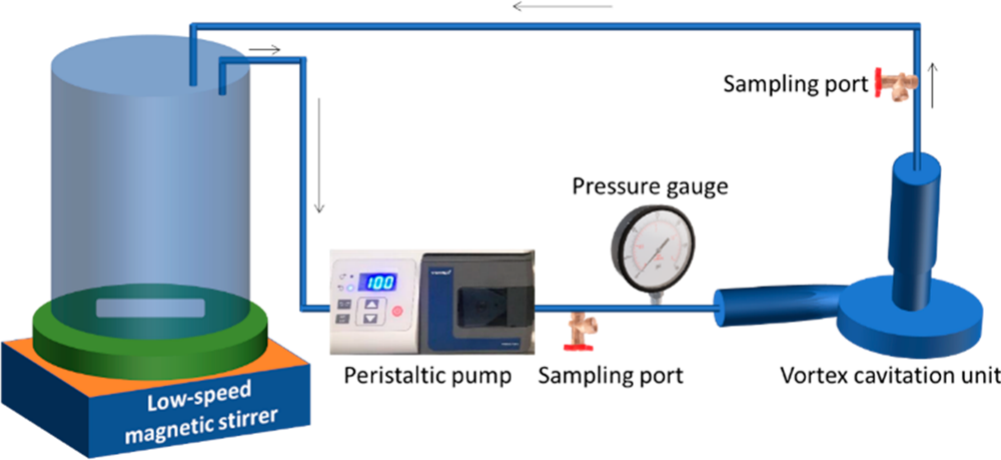
Schematic of multiple pass vortex-based hydrodynamic cavitation experimental setup.

The multiple pass experiments were performed for generating liquid–liquid emulsions using vortex-based HC unit (based on the design of Vivira Process Technologies and the patent of Ranade et al.^28^). Two scales of HC devices (throat diameter, *d*_T_ = 3 mm, nominal capacity 1 LPM and *d*_*T*_ = 12 mm, nominal capacity, 20 LPM) were used in the present work to examine the effect of scale-up on the emulsion characteristics. The schematic of the experimental setup is shown in Figure 1. The typical photographs of both the experimental set-ups are shown in Figures S1 and S2 of the Supporting Information. The detailed dimensions of HC units with reference to the throat diameter were the same as reported by Simpson and Ranade.^29^

In the present study, liquid–liquid emulsions were generated using two different oils: rapeseed oil (RO, *ρ*_*O*_ = 915 kg/m^3^, *μ*_*O*_ = 6.2 × 10^–2^ Pa·s, sourced from Newgrange Gold, Tesco, Ireland) in demineralized water (*ρ*_*A*_ = 997 kg/m^3^, *μ*_*A*_ = 7.972 × 10^–4^ Pa·s, sourced from Elga ultrapure water system) and tetrachloroethylene (TCE) oil (purity 99.5%, *ρ*_*O*_ = 1620 kg/m^3^, *μ*_*O*_ = 6.0 × 10^–2^ Pa·s, sourced from Honeywell) in demineralized water. The RO–water based emulsions are widely used in food industry and TCE–water based emulsions primarily used in dry cleaning applications of fabrics. Preliminary experiments were carried out with an RO system with different concentrations of TWEEN 20 surfactant and found that the emulsion was stable for at least for 74 days without coalescence with 2 wt % surfactant of the total weight of the emulsion.^25^ Therefore, 2 wt % of TWEEN 20 (sourced from MP Biomedicals, LLC, France) surfactant was used for the RO in water system to prevent drop coalescence. For TCE in water system, 1 wt % of oil-soluble surfactant (SPAN80) and a minimal amount of (0.002 wt %) of water-soluble surfactant (CTAB) were added to the demineralized water. The experiments were performed with total volume of 300 and 5000 mL for a small scale (*d*_*T*_ = 3 mm) and large scale (*d*_*T*_ = 12 mm) devices, respectively. The oil-in-water pre-emulsion was generated by adding 5 and 15% of oil volume in water and using a magnetic stirrer (Fisherbrand) operated at 300 rpm for 10 min. A negligible difference was found in DSD beyond 10 min.^25^ Therefore, after stirring for 10 min, the pre-emulsion was pumped through the vortex-based cavitation unit using the peristaltic pump for a small-scale system (Longer BT600–2J). For a large-scale system, the progressive cavity pump (Roto flow, MCCH011J2CD1Y) was used to pump the pre-emulsion through the HC device.

These experiments were carried out by setting the device at the desired pressure drop using a variable frequency drive. Experiments were carried out up to ∼200 passes through cavitation device (number of passes through cavitation device, *n*_*p*_ = *Qt*/*V* where *Q* is flow rate through cavitation device, *V* is the volume of emulsion in the experimental loop including piping, device, and holding tank, and *t* is flow time. The influence of pressure drop across cavitation device on droplet breakage and resulting droplet size distribution was investigated by setting the pressure drop across the cavitation device from 50 to 250 kPa with intervals of 100 kPa. In the vortex-based HC device, the cavitation inception occurs between Δ*P* = 50 and 80 kPa.^30^ Therefore, the pressure drop of 50 kPa was considered the lowest pressure drop to perform controlled experiments. However, at 50 kPa, the cavitation occurred intermittently and resulted in bimodel DSD. Detailed discussion on effect of pressure is provided in section 4.2. The time required to circulate the total volume of system (300 mL) for one time (1 pass) is ∼14 s. First 5 passes were ignored to avoid start-up effects. A total of 5 samples at 15, 35, 55, 105, and 205 passes were collected from the holding tank. The collected samples were analyzed using a Malvern MasterSizer 3000. The refractive index for RO and TCE was set to 1.466 and 1.5, respectively, for the lasers [red laser (632.8 nm) and blue laser (470 nm)]. Water was used as a dispersant medium (water) at room temperature (20 °C). The experiments of different oil systems (RO–water and TCE–water) were performed three times to quantify error bars. The error bars on measured DSD and Sauter mean diameter values are included wherever possible.

**Figure 2:**
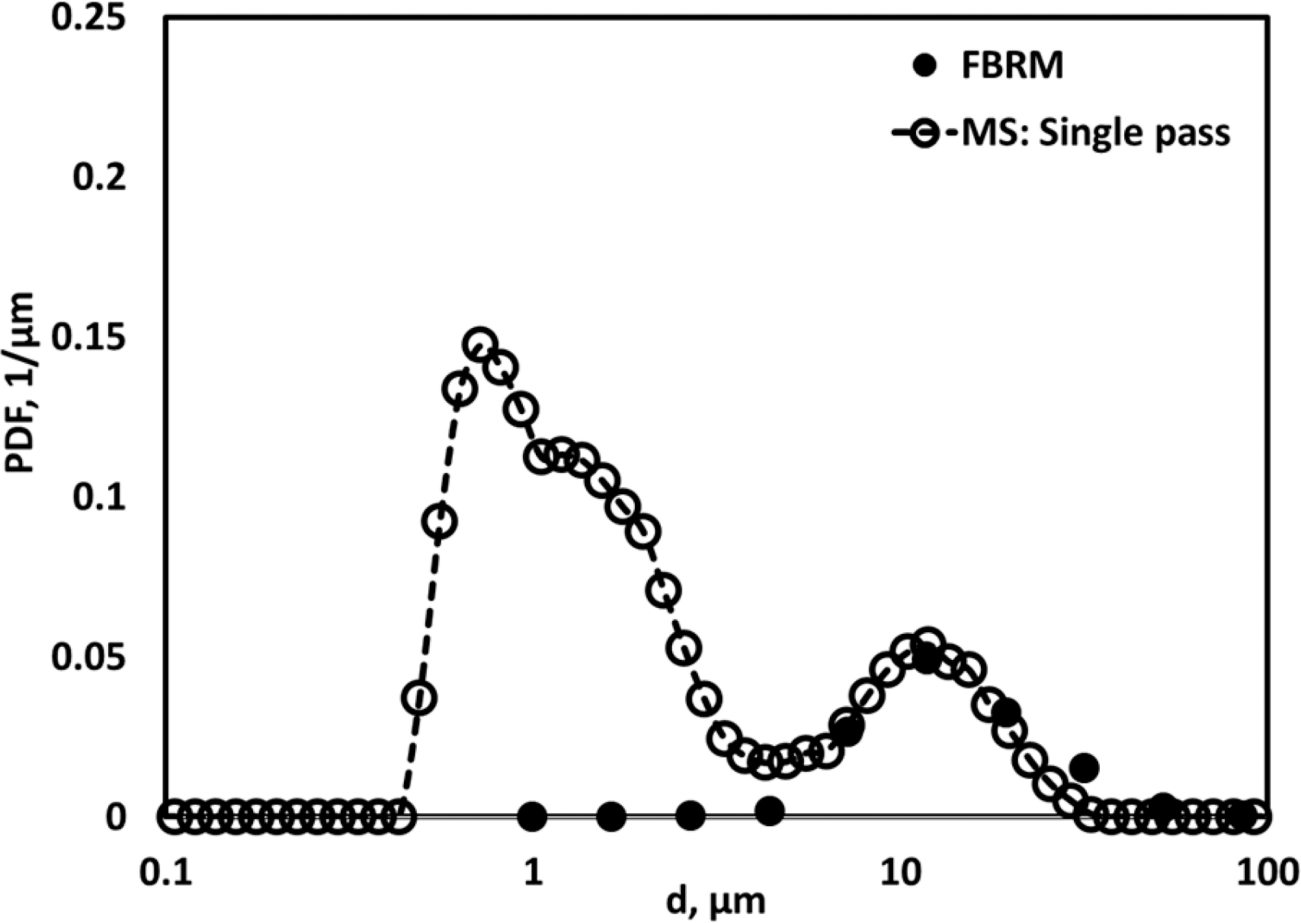
Drop size distribution measurement through MS and FBRM (FBRM data is taken from ref (25)) (Δ*P* = 250 kPa, *v*_*t*_ = 3.2 m/s, *α*_*O*_ = 0.05).

As mentioned in section 1, in our previous study, with a single pass through HC device, we obtained monomodel DSDs as measured by FBRM. Ramisetty et al.^14^ performed similar experiments for producing emulsion using venturi type HC devices and found bimodel distribution. Highly localized intense shear and energy dissipation rates expected in cavitation devices apparently lead to submicrometer size droplets which were missed in our previous work. We therefore repeated the analysis of DSD of emulsions obtained via single pass through HC device using MS. As per our expectation, DSD obtained through MS showed bimodel distribution. We have compared the DSD obtained through MS with the DSD analyzed using FBRM in Figure 2. It was satisfying to see that peak in DSD with a larger mean diameter obtained from MS measurements (at ∼13 μm) almost overlapped with DSD obtained with FBRM (see Figure 2). In addition, MS measurements showed another peak in DSD with submicron mean (at ∼0.9 μm). Considering the ability of the MS to capture smaller size droplets, the MS was used in the present study for multiple pass HC treatment of the emulsions.

## 3 Computational Model

Recently, Thaker and Ranade^25^ modeled the flow characteristics of liquid–liquid emulsion in the presence of cavitation in the vortex-based HC device. In that work,^25^ we used a mixture model approach coupled with PBM to simulate cavitating gas–liquid–liquid flow and simulated drop breakage and drop size distribution generated in a single pass through HC device. In this work, we have extended our previous model to simulate the influence of multiple passes through the vortex-based HC device on DSD. The details of model equations, boundary conditions, and numerical and population balance equations (PBEs) solution methodologies used in the present work are provided in section 1 of the Supporting Information.

In our previous work,^26^ we carried out simulations using two approaches. The coupled simulations (transient simulations with simultaneous solution of flow equation and population balance equations) and the decoupled simulation (by solving only population balance equations after establishing of the flow). The difference between both approaches was insignificant; therefore, the decoupled approach was used in the present work for simulating the influence of multiple passes on DSD. The flow equations were solved at least for the three residence times for ensuring adequate convergence. The sensitivity of time step to solve PBEs was performed and found that the results were not sensitive to further reduction in the time step below 0.01 s. The time step of 0.01 s (which is 0.05 times residence time) was used in all the subsequent simulations. The minimum and maximum drop sizes of the representative group were kept at 0.01 and 1000 μm, respectively. The influence of the number of groups used to represent DSD was quantified by performing simulations with 20, 40, and 80 groups of drop sizes. These results are shown in Figure S4 of the Supporting Information. A marginal difference in simulated DSD was found in the results obtained with 20 and 40 groups. Therefore, 20 groups were considered for all subsequent simulations of DSD. For simulating multiple passes, it was assumed that droplet breakage does not occur outside the HC device and the DSD at the outlet is specified as inlet boundary condition. The volume fraction of each of the groups of drop sizes at the inlet was specified by calculating mass averaged volume fraction of the corresponding group at the outlet as
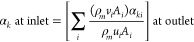
1where *ρ*_*m*_ is mixture density, *v*_*i*_ and *A*_*i*_ are velocity of the mixture in cell *i* and area of cell *i*, respectively, and *α*_*ki*_ is the volume fraction of *k*th size group in cell *i* of outlet. The summation sign indicates summing over all the computational cells of the outlet. After solving the PBEs for three residence times, the data files generated for each group through monitors (at the outlet) were set as inlet group fractions at inlet boundaries as mentioned in eq 1. The simulated DSD results are discussed in section 4.6. All the simulations were carried out using Ansys Fluent 2020.

## 4 Results and Discussion

### 4.1 Effect of Multiple Passes on Drop Size Distributions

**Figure 3:**
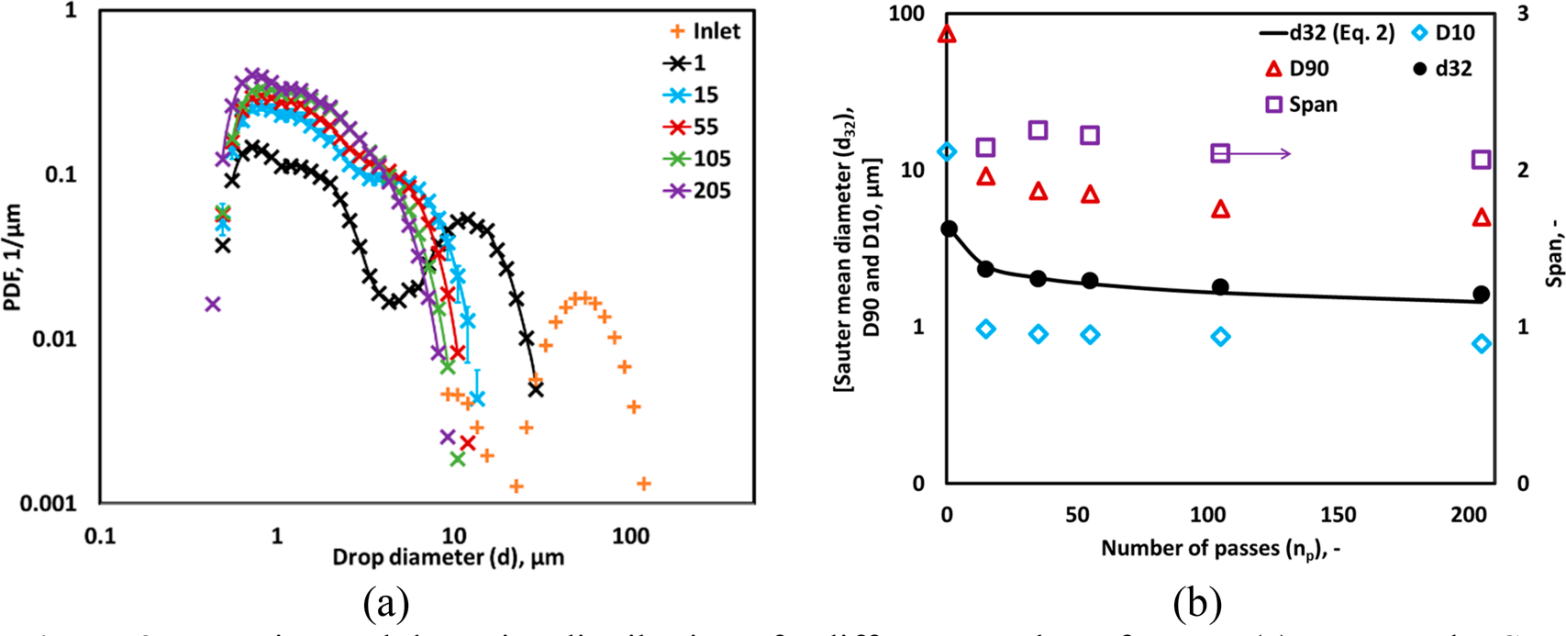
Experimental drop size distribution of a different number of passes. (a) Measured DSD; lines indicate an overall trend and (b) *D*10, *D*90, Sauter mean diameter, and relative span. The error bars of Sauter mean diameter are smaller than the symbol size (*d*_*T*_ = 3 mm, Δ*P* = 250 kPa, *v*_*T*_ = 2.95 m/s, *α*_*O*_ = 0.05).

The preliminary experiments were carried out to characterize flow of oil–water emulsion versus pressure drop behavior of the vortex-based HC device for oil volume fraction up to 15%. The Euler number  was found to be 50 irrespective of oil volume fraction, which is very close to the value of *Eu* reported by Simpson and Ranade^34^ for cases without oil. As a base case, the influence of number of passes through the cavitation device on resulting DSD was investigated for pressure drop of 250 kPa. The influence of number of passes on DSD is shown in Figure 3a. A single pass through the cavitation device results in the bimodal distribution of DSD. The larger peak of DSD was found to decrease with an increase in the number of passes. Chatzi and Kiparissides^31^ mentioned that the production of bimodal DSD is mainly due to the intense breakage involving the formation of small satellite drops during the breakage of parent droplets. Janssen et al.^32^ mentioned that before the breakage, the parent droplet might be stretched into threads and produce a large population of smaller drops. Our previous CFD simulations indicated that a small cavitating core exists in the HC device used in this work. Therefore, in a single pass through HC device, only some fraction of the droplets of pre-emulsion may pass through the cavitating core and encounter collapsing cavities.^25^ The other fraction of droplets may not encounter collapsing cavities and get broken because of prevailing turbulence and shear in the HC device besides cavitating core region. The data shown in Figure 3a indicate that the droplets not encountering collapsing cavities are broken down to droplets of ∼10^1^ μm. There is a finite fraction of droplets encountering collapsing cavities which are broken down to ∼10° μm. These two different droplet mechanisms lead to bimodal nature of the resulting DSD. The possible reason behind the bimodel DSD and the role of cavitation in droplet breakage are discussed again in section 4.6. As the number of passes increase, there is more and more chance that droplets encounter collapsing cavities and therefore eventually bimodal nature of DSD vanished beyond 55 passes through HC device (see Figure 3a). Key commonly used characteristics like Sauter mean diameter, *D*10, *D*90, and span (see notations for definitions) are calculated from the measured DSD. The influence of number of passes on these characteristics is shown in Figure 3b. *d*_32_ was found to be 5.7 μm after a single pass and decreased to 2.5 and 1 after 15 and 205 passes, respectively. The larger droplets in fact are reduced significantly within first 15 passes which can be seen from reduction of *D*90 from 74 to 9.2 μm in 15 passes. The variation in Sauter mean diameter, *d*_32_, as a function of number of passes may be represented as
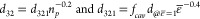
2where, *n*_*p*_ is number of passes, *d*_321_ is a Sauter mean diameter after a single pass through the HC device, *f*_*cav*_ is a parameter that represents the effect of cavitation, ε̅ is mean energy dissipation rate in the HC device, and *d*_@ε̅=1_ is a Sauter mean diameter at ε̅ = 1 m^2^/s^3^. *f*_*cav*_ is a lumped parameter representing influence of cavitation, and it will change with the device design, scale, and operating conditions since these will influence number density of generated cavities, intensity of cavity collapse, and probability of collision among cavities and oil droplets. For devices without cavitation or HC devices operated below cavitation inception, the value of *f*_*cav*_ becomes 1, and the drop breakage occurs because of the mean turbulent energy dissipation rate, ε̅, in the device. In the present study, the values of *f*_*cav*_ were found to be 0.8 and 0.5, respectively, for lab-scale (*d*_*T*_ = 3 mm) and bench-scale (*d*_*T*_ = 12 mm) devices. *d*_@ε̅=1_ is a parameter related to physical properties of oil and changes with oil–water systems. In the present study, the values of *d*_@ε̅=1_ were found to be 200 and 66 for the RO–water and TCE–water systems, respectively. The average energy dissipation rate per unit mass (ε̅) of vortex chamber may be calculated from pressure drop (Δ*P*) and flow rate (*Q*) through the device as
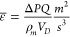
3where *V*_*D*_ is the volume of HC device and *ρ*_*m*_ is the mixture density.

Further, to examine the performance of vortex-based HC device, breakage efficiency (η) was estimated. η can be determined from the *d*_32_ and the interfacial area (*A*) by
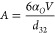
4The net surface area generation, *A*_*net*_ (m^2^), at number of passes equal to *n*_*p*_, can be written as
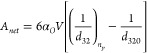
5where *d*_320_ is a Sauter mean diameter of pre-emulsion (at zeroth pass through HC device). By considering the new surface area generation and interfacial tension, the theoretical minimum energy required for drop breakage may be calculated as
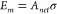
6The efficiency of emulsification can therefore be determined by considering a ratio of minimum energy dissipation and actual energy dissipation as^33,34^
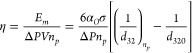
7It can be seen from eqs 2 and 7 that the efficiency will decrease with an increase in number of passes. Therefore, for comparison purposes, efficiency values for a single pass (*n*_*p*_ = 1) were considered. The calculated η of a single pass for 250 kPa was 0.7%. It is useful to compare the η of different operating and geometric conditions (i.e., Δ*P*, *α*_*O*_, device scale, and oil systems) for single pass and for multiple passes based on energy consumption per unit mass of emulsion, *E*, which may be related to pressure drop as
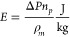
8where *ρ*_*m*_ is mixture density. The value of *E* for 250 kPa was found to be 0.26 kJ/kg which is significantly lower than conventional devices. The influence of pressure drop, device scale, and type of oil on η and comparison with other published studies are discussed in the following sections.

### 4.2 Effect of Pressure Drop

**Figure 4:**
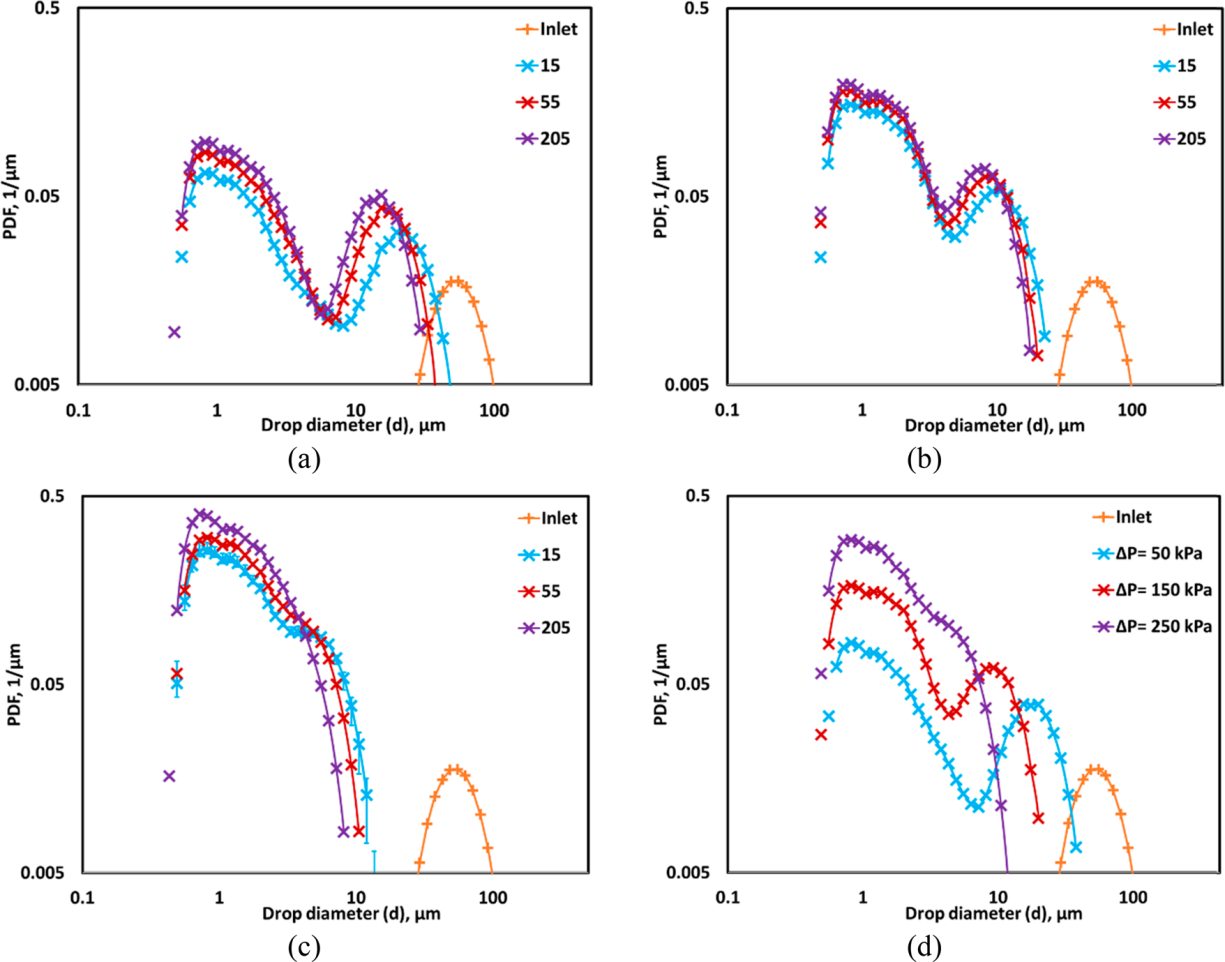
Measured drop size distribution for different Δ*P* = (a) 50 kPa, (b) 150 kPa, and (c) 250 kPa. (d) Comparison of DSD for different Δ*P* at *n*_*P*_ of 35 passes. (*α*_*O*_ = 0.05; *d*_*T*_ = 3 mm).

**Figure 5:**
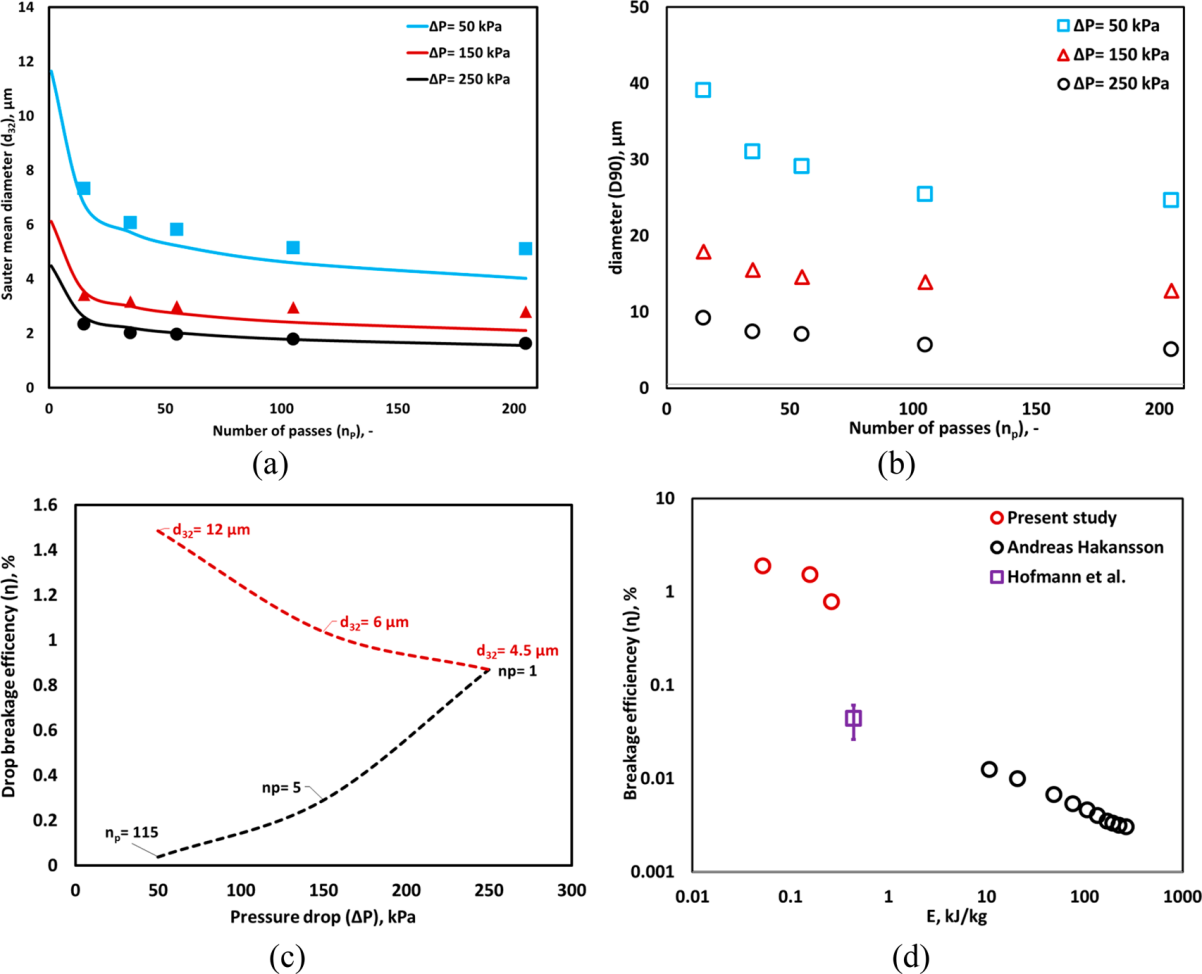
(a) Measured and calculated *d*_32_ (eq 2) for different pressure drop (Δ*P* = 250, 150, and 50 kPa corresponding ε̅ = 7600, 3500, and 700 m^2^/s^3^, respectively) and number of passes. (b) Measured *D*90, for different Δ*P* (c) breakage efficiency as a function of pressure drop for different drop size and number of passes estimated from eq 2 (red dotted line is for *n*_*p*_ = 1 and black dotted line is for *d*_32_ = 4.5 μm; lines indicate an overall trend). (d) Comparison of droplet breakage efficiency as a function of energy consumption per unit mass of emulsion *E* between the high-pressure homogenizer (ref (31)) and fractal multipliers (ref (32)) and vortex-based HC device for a single pass treatment. (*d*_*T*_ = 3 mm).

Multiple-pass experiments were performed with different inlet pressure across the device, i.e., Δ*P* = 50 and 150 kPa, to analyze the influence of pressure drop on droplet breakage. The measured DSDs at different pressure drops across the HC device are shown in Figure 4a–c. Unlike the case of Δ*P* = 250 kPa, the bimodel nature of DSD for lower pressure drop cases (Δ*P* = 50 and 150 kPa) persisted for a much higher number of passes. For the case of lowest pressure drop (50 kPa), the bimodel nature of DSD was observed even after 205 passes (see Figure 4a). As mentioned in section 2.1, at Δ*P* = 50 kPa, cavitation inception occurs intermittently. The data shows some droplets breaking down to submicron scale which is unlikely in the absence of cavitation. The influence of pressure drop on key characteristics like *d*_32_ and *D*90 is shown in Figure 5a,b. It can be seen that increase in pressure drop leads to reduced values of *d*_32_ and *D*90,which is in line with intuitive understanding. With an increase in inlet pressure drop, the power dissipated on the diode (Δ*P* × *Q*) was increased from 0.5 to 5.48 W for 50 to 250 kPa, respectively. The pressure drop across the device, therefore, is an important process parameter controlling *d*_32_ and other characteristics (see Figure 5a,b). The values of *D*10, *D*50, and *D*90 for different Δ*P* are provided in Table S1 of the Supporting Information. Compared to the lower pressure drops, the drop size reduced rapidly, and DSD was found to be in the range of 1–10 μm after 35 passes at Δ*P* = 250 kPa (see Figure 4d). The observed trends of *d*_32_ for different Δ*P* and *n*_*p*_ were well-represented in terms of ε̅ using eq 2, as shown in Figure 5a.

The breakage efficiency (η) was calculated using eq 7 and compared for different Δ*P* at the same *d*_32_ and *n*_*p*_. To estimate the similar value of *d*_32_ (*d*_32_= 4.5 μm) for different Δ*P* cases, the *n*_*p*_ was extrapolated using eq 2. For achieving the Sauter mean diameter of 4.5 μm, the values of *n*_*p*_ were found to be 1, 5, and 115 for Δ*P* = 250, 150, and 50 kPa, respectively. Figure 5c shows the η for different Δ*P* at *d*_32_= 4.5 μm. The η was increased with an increase in Δ*P* and showed a maximum value (η = 0.9%) at 250 kPa due to the requirement of lower number of passes to generate small drop size. On the other hand, η was found to be higher at a lower Δ*P* for a single pass because the actual energy dissipated to break the drops was lower at lower Δ*P* (see Figure 5c). Note that the Sauter mean diameter obtained at a lower pressure drop (50 kPa) was 12 μm after a single pass which is much larger than the value obtained after a single pass at 250 kPa (4.5 μm). This information is useful to select appropriate operating condition for different applications based on the requirement of final drop size to get better η of the device.

Further, the η for different Δ*P* were compared with the η determined by Andreas Hakansson^35^ and Hofmann et al.^36^ for the emulsification process using a high-pressure homogenizer system and fractal multiplier, respectively. For comparison, the values of *α*_*O*_ was set to be 0.01 in eq 7 as considered by Andreas Hakansson.^35^ In our previous study, we found that the influence of oil volume fraction on drop size from 0.01 to 0.05 was marginal in a vortex-based HC device.^25^ Therefore, the drop size for volume fraction at 0.01 was considered to be the same as that measured for 0.05 in the present work. Figure 5d shows the comparison of η at different *E* between the high-pressure homoginizer^35^ and vortex-based HC device for a single pass. The η of the vortex-based HC device showed significantly higher values compared to the high-pressure homogenizer. Hofmann et al. performed emulsification experiments in fractal multiplier using different internals in microchannels^36^ and calculated the η for *α*_*O*_ of 0.6 at *E* of 0.44 kJ/kg. For comparison, η was estimated for *α*_*O*_ of 0.01 and found to be in the range of 0.02–0.09. This indicates that vortex-based HC device provides an excellent drop breakage efficiency at significantly lower energy consumption than high-pressure homogenizer. The influence of *α*_*O*_ on the DSD, drop diameters, and η for different *n*_*p*_ is discussed in the following section.

### 4.3 Influence of Oil Volume Fraction

**Figure 6:**
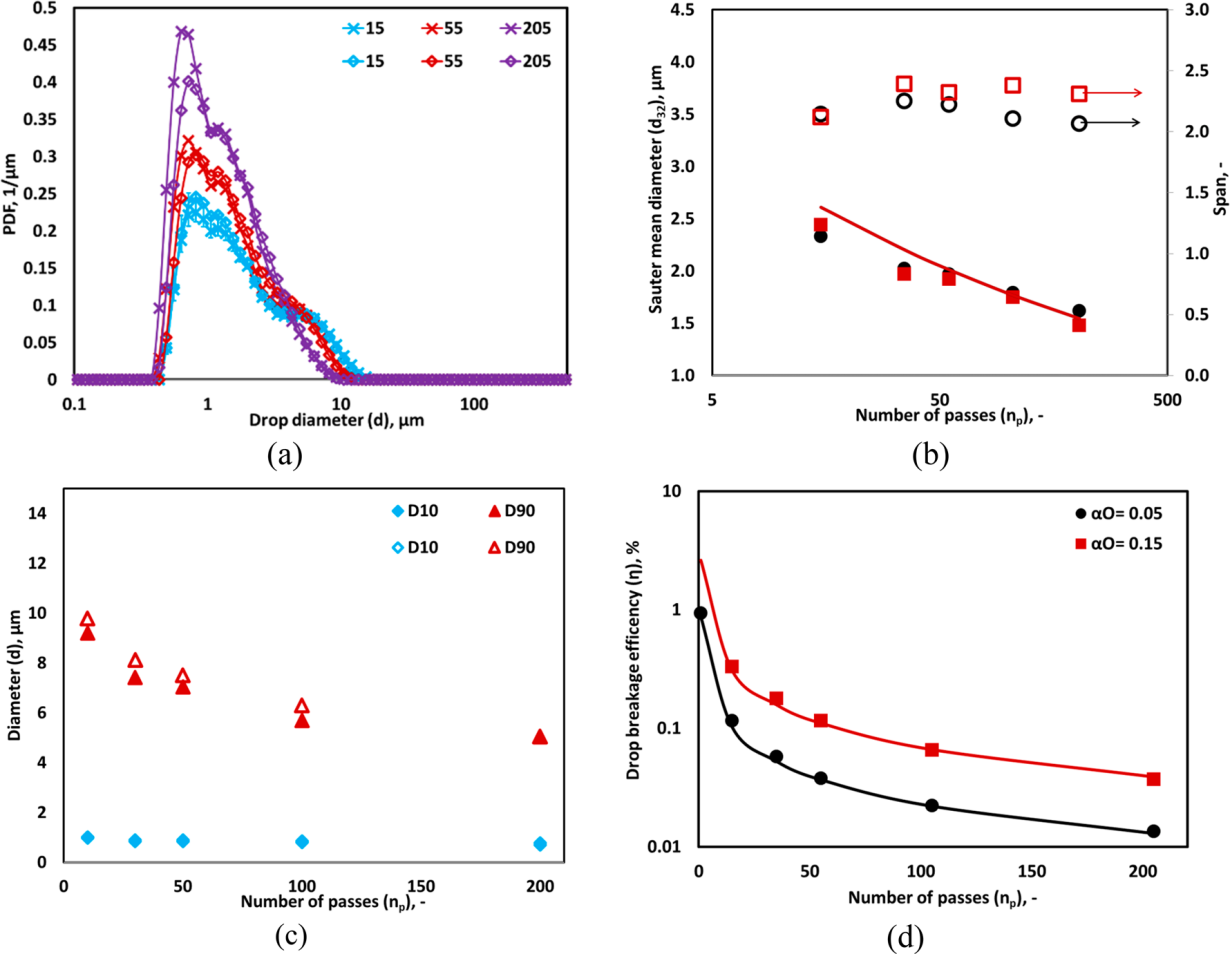
Comparison between drop size distributions measured for 5% (× symbol) and 15% (◊ symbol) oil volumes. (a) Measured DSD; lines indicate an overall trend. (b) Sauter mean diameter, *d*_32_ for *α*_*O*_ of 0.05 (● symbol) and 0.15 (■ symbol) and span; lines indicate predicted *d*_32_ using eq 2. (c) *D*10 and *D*90, for *α*_*O*_ of 0.05 (filled symbol) and 0.15 (hollow symbol). (d) Drop breakage efficiency for different number of passes (*d*_*T*_ = 3 mm, Δ*P* = 250 kPa, *v*_*t*_ = 2.95 m/s).

The multiple pass experiments were performed to examine the effect of oil volume fraction, *α*_*O*_ on the DSD, *d*_32_ and η at Δ*P* = 250 kPa. Figure 6a shows the comparison of measured DSD for different oil volume fractions, i.e., 0.05 and 0.15. The difference in DSD was insignificant between the *α*_*O*_ of 0.05 and 0.15, except for the peak values of 205 passes. A marginal difference was found in *d*_32_ between *α*_*O*_ of 0.05 and 0.15 (see Figure 6b). This is reflected in the *D*90 profile (see Figure 6c). A similar trend was also observed by Ramisetty et al.^37^ in the case of the emulsification process in the linear flow HC device. The differences in *D*10 and *D*50 were not significant as the mean and minimum drop size range in DSD for *α*_*O*_ of 0.15 was the same as that obtained in *α*_*O*_ of 0.05. The η at *α*_*O*_ of 0.05 and 0.15 for different numbers of passes is shown in Figure 6d. As expected, the η increases significantly with an increase in *α*_*O*_ and reached up to 2.5% at *α*_*O*_ of 0.15 because the marginal increase in *d*_32_ compensated adequately with *α*_*O*_ (see Figure 6d). The difference in η was found to be significant between *α*_*O*_ of 0.05 and 0.15 at even 205 passes. This indicates that the influence of *α*_*O*_ on η was more as compared to the drop size. The effect of different liquid–liquid systems on DSD, diameters, and η is discussed in the following section.

### 4.4 Comparison of Different Oil Systems

**Figure 7:**
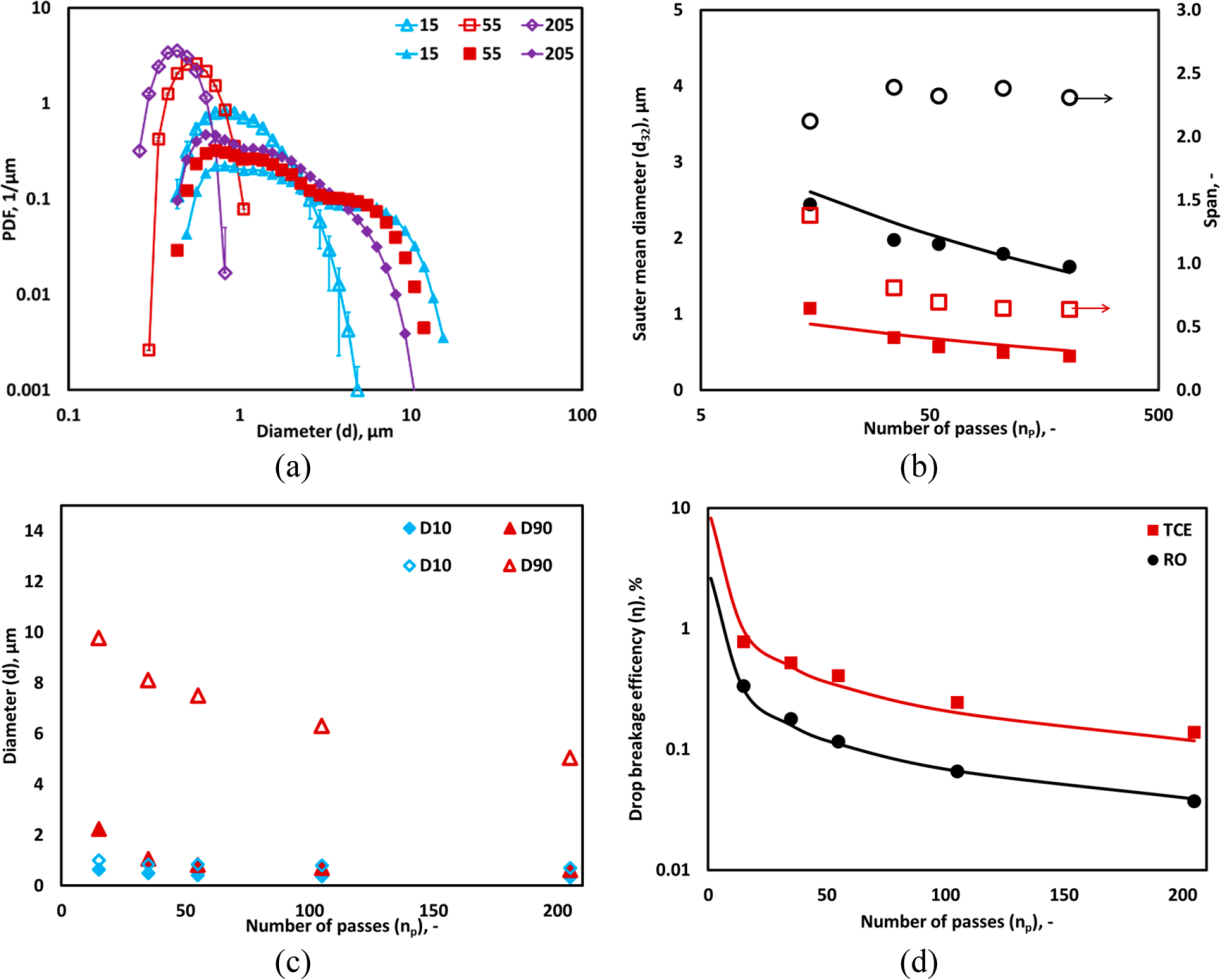
Comparison between the drop size distributions measured for different oil systems; RO (× symbol) and TCE (◊ symbol). (a) Measured DSD; lines indicate an overall trend. (b) Sauter mean diameter, *d*_32_ for RO (● symbol) and TCE (■ symbol)and span; line represents the predicted *d*_32_ using eq 2. (c) *D*10 and *D*90 for RO (hollow symbol) and TCE (filled symbol). (d) Drop breakage efficiency of RO–water and TCE–water systems. The symbol denotes measured values and lines indicate breakage efficiency calculated by *d*_32_ obtained from correlation (eq 2) (*d*_*T*_ = 3 mm, Δ*P* = 250 kPa, α_0_= 0.15 *v*_*t*_ = 2.95 m/s).

To investigate the influence of different oil–water systems on DSD and diameters of emulsion, the experiments were carried out with a TCE–water system for *α*_*O*_ of 0.15 using lab-scale experimental setup. Figure 7a shows the comparison of DSD and diameter between the RO–water and TCE–water systems. The interfacial tension of both systems without surfactant is similar (35 mN/m).^38^ It can be seen that the DSD of TCE shows a monomodel distribution at the different number of passes (Figure 7a). The drop size of TCE was found to be significantly lower than RO. In the TCE–water system, in addition to intense shear by collapsing cavities, the turbulence shear generated due to convective flow may also play an important role in reducing drop size significantly. Therefore, after only 15 passes *d*_32_ and *D*90 were found to be <1 μm (see Figure 7b,c). The trend of *d*_32_ for the TCE–water system can also be represented using eq 2. As mentioned earlier in section 4.1, the value of *d*_@ε̅=1_ was found to be 66 for the TCE–water system in eq 2. The span of the RO–water system showed a constant trend after 15 passes (see Figure 7b); therefore, the difference between *D*90 and *D*10 was constant at different numbers of passes (see Figure 7c). On the other hand, in the TCE–water system, due to the decrease in span for different number of passes, the differences between *D*90 and *D*10 were found to reduce and become marginal after 35 passes (see Figure 7c). The fundamental reasons for different trends and nature of DSD and diameters are not yet fully understood, and microscale investigations are required to investigate the potential effect of different surfactants to determine the nature of DSDs for different liquid–liquid systems. Figure 7d shows the drop breakage efficiency (η). As expected, due to the lower *d*_32_ in the TCE–water system than that in the RO–water system, the net surface area was found to be substantially higher for the TCE–water system. As a result, the η profile for the TCE–water system showed higher values for a different number of passes than the RO–water system. The influence of device scales on the DSD, drop diameters, and the comparison of η with literature for different *n*_*p*_ is discussed in the following section.

### 4.5 Effect of Device Scales

**Figure 8:**
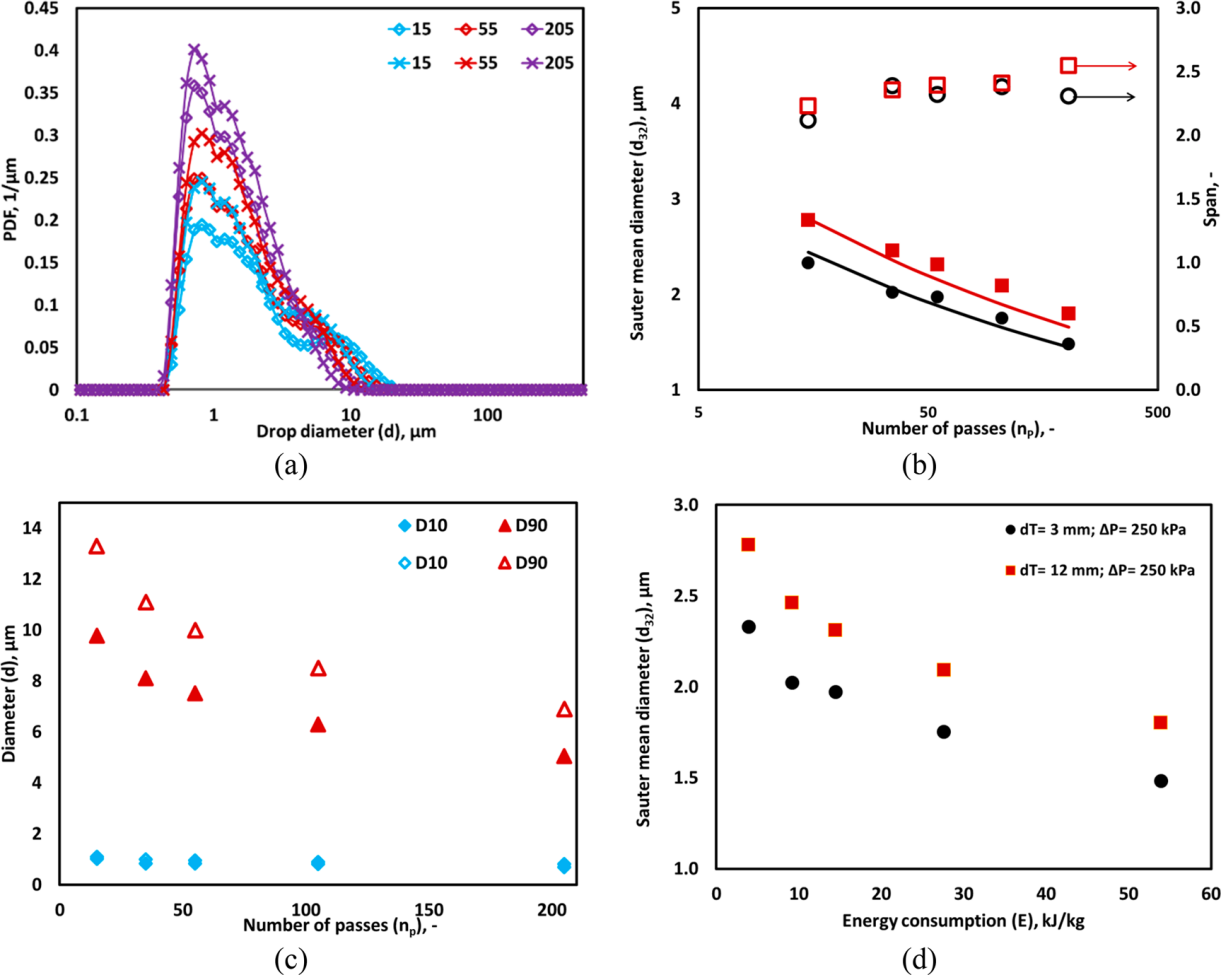
Comparison between the drop size distributions obtained through different scales of vortex diode; throat diameter (*d*_*T*_) of 3 mm (× symbol) and 12 mm (◊ symbol). (a) Measured DSD; lines indicate an overall trend. (b) Sauter mean diameter, *d*_32_ for *d*_*T*_ of 3 mm (• symbol) and 12 mm (■ symbol) and relative span; lines indicate predicted *d*_32_ using eq 2 and relative span. (c) *D*10 and *D*90 for *d*_*T*_ of 3 mm (filled symbol) and 12 mm (hollow symbol). (d) *d*_32_ with energy consumption for different device scales (*α*_*O*_ = 0.05, Δ*P* = 250 kPa, *v*_*t*_ = 2.95 m/s).

**Figure 9:**
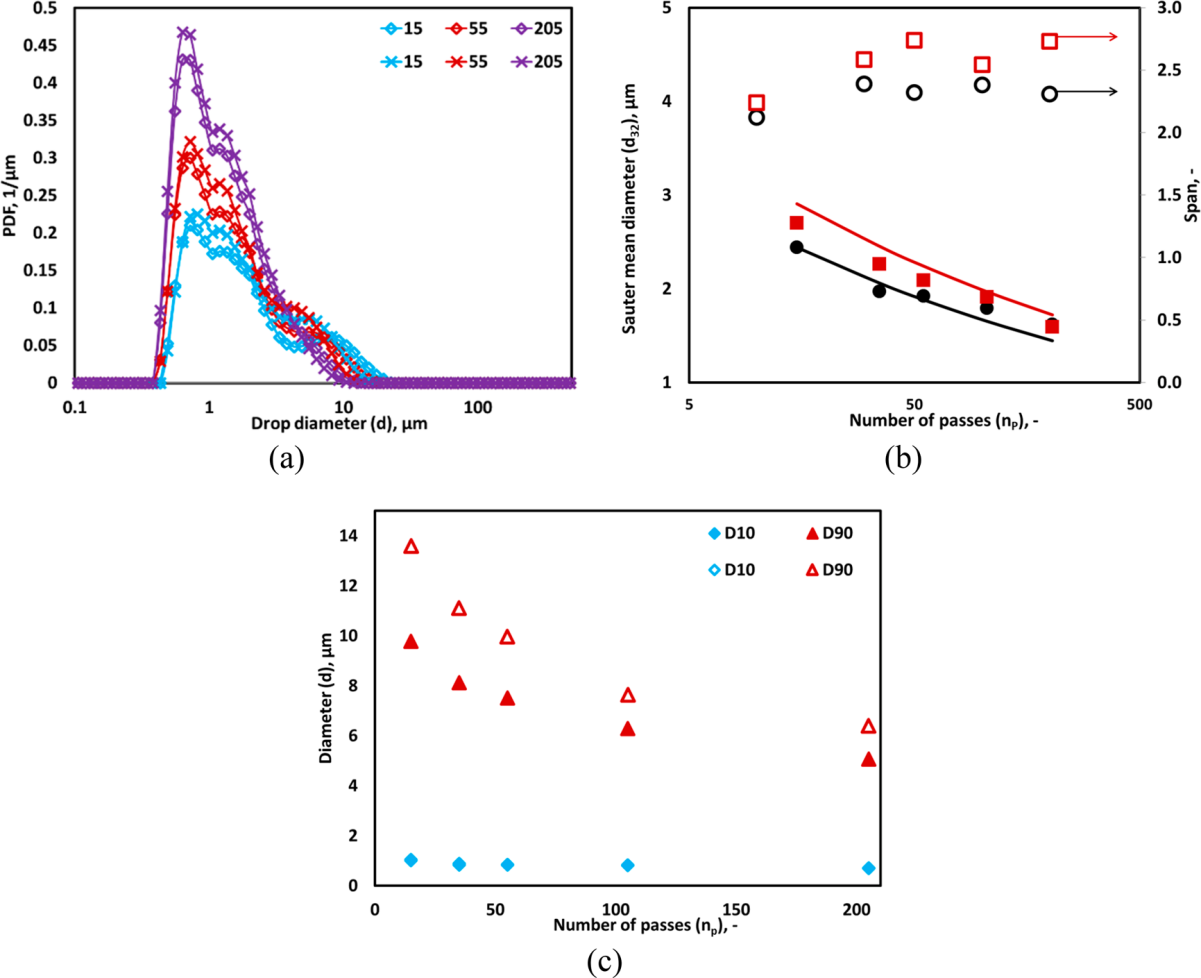
Comparison between the drop size distributions obtained through different scales of vortex diode; throat diameter (*d*_*T*_) of 3 mm (× symbol) and 12 mm (◊ symbol). (a) Measured DSD; lines indicate an overall trend. (b) Sauter mean diameter, *d*_32_ for *d*_*T*_ of 3 mm (● symbol) and 12 mm (■ symbol) and relative span; lines indicate predicted *d*_32_ using eq 2. (c) *D*10 and *D*90 for *d*_*T*_ of 3 mm (filled symbol) and 12 mm (hollow symbol), (*α*_*O*_ = 0.15, Δ*P* = 250 kPa, *v*_*t*_ = 2.95 m/s).

It is important to quantitatively understand the influence of device scale on the DSD for scale-up of the device. Therefore, the experiments were performed using a bench-scale device (*d*_*T*_ = 12 mm; *Q* = 20 LPM) with different oil volume fractions. Figures 8 and 9 show the DSD, *d*_32_, *D*90, and *D*10 for *d*_*T*_ of 3 mm (lab-scale device; capacity: 1 LPM) and 12 mm (bench-scale device; capacity 20 LPM), respectively. A significant difference was found in DSD between the lab-scale and bench-scale devices for all *α*_*O*_ considered in the present work (see Figures 8a and 9a). The Sauter mean diameters obtained with these two devices and other key characteristics of observed DSD are shown in Figure 8b,c. The observed trends of *d*_32_ were represented using eq 2. The measured volumes (*V*_*D*_) of lab-scale and bench-scale devices were 0.7 and 45 mL, respectively. Both the devices were operated at 250 kPa pressure drop and the corresponding ε̅ of both the devices were 7600 and 1800 m^2^/s^3^, respectively. The values of *f*_*cav*_ for lab-scale and bench-scale devices were 0.8 and 0.5, respectively, in eq 2. Considering the substantially lower value of ε̅ in the larger device, one would expect the Sauter mean diameter with the larger device to be almost 1.8 times of that observed with the smaller device. However, because of the presence of cavitation which generates smaller droplets and dominance of smaller droplets in determining the Sauter mean diameter, the observed value of the Sauter mean diameter with the larger device (12 mm) is only about 1.1 times larger than with the smaller device (3 mm). This is reflected in the smaller value of *f*_*cav*_ for the larger device. The complex interactions of drop breakage due to turbulence flow field in the device and collapsing cavities generate bimodal DSD at lower passes. It can be seen from Figure 8b,c that the drop size obtained after 15 passes (*D*90 = 9.78 μm; *d*_32_= 2.3 μm) in the lab-scale device was found in the bench-scale device after 55 passes (*D*90 = 10 μm; *d*_32_= 2.3 μm). Similarly, the energy consumption (*E*) of the bench-scale device was 14.5 kJ/kg for *d*_32_ of 2.3 μm, whereas, for the lab-scale device it was 4 kJ/kg for same value of *d*_32_ (see Figure 8d). In both the device scales, *d*_*min*_ was ∼1 μm (see Figures 8a and 9a), therefore, the difference in *D*10 was insignificant between both the devices (see Figures 8c and 9c).

The extent of influence of oil volume fraction on drop size was found to decrease with an increase in the number of passes due to the higher exposure of droplets with intense turbulence zones developed at the vortex core. The differences in DSD and diameters between both the device scales were found to decrease with an increase in oil volume fraction (*α*_*O*_) from 0.05 to 0.15 (see Figures 8 and 9). In the lab-scale device, the difference in *d*_32_ between *α*_*O*_ of 0.05 and 0.15 was less than 0.1 μm after 35 passes, whereas, it was found to be 0.2 μm after 35 passes in the bench-scale device. In the bench-scale device, the closer presence of other oil droplets may also influence the nature and intensity of cavitation.^14,39^ Therefore, in multiple passes of HC treatment, *d*_32_ was found to decrease more at higher *α*_*O*_ in the bench-scale device as compared to the lab-scale device with the number of passes.

**Figure 10:**
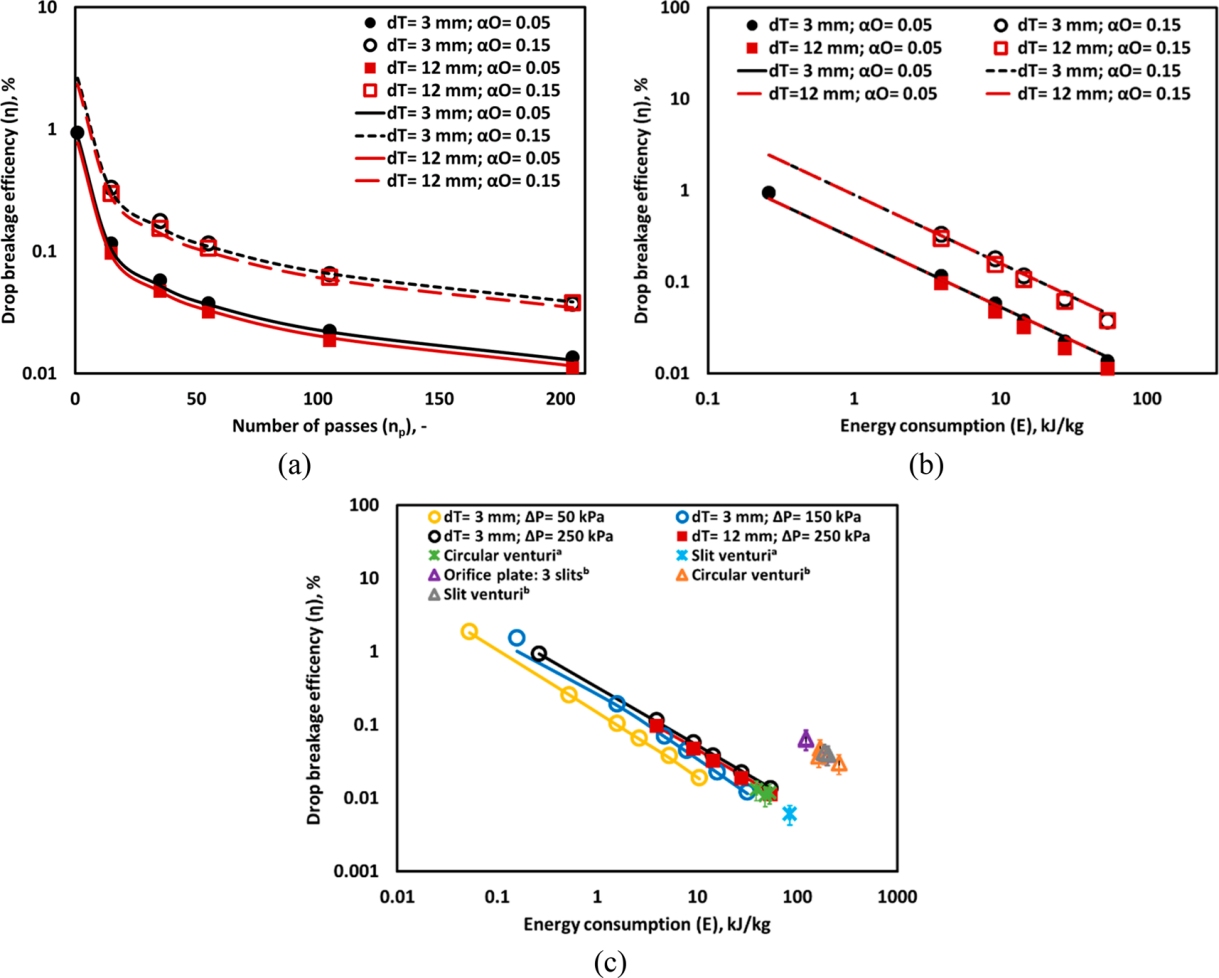
Drop breakage efficiency of (a) different number of passes and (b) breakage efficiency as a function of energy consumption, Δ*P* = 250 kPa; lines indicate predicted η using eq 9. (c) Energy consumption at *α*_*O*_ of 0.05 (The symbol denotes measured values and lines indicate breakage efficiency calculated by *d*_32_ obtained from correlation (eq 2); (refs for superscripts a and b are (11) and (16), respectively).

Further, the breakage efficiencies for both the device scales were calculated using eq 7. Comparison of η between the *α*_*O*_ of 0.05 and 0.15 was made between the experimental data and predicted values from eq 2 for the different number of passes, as shown in Figure 10a. As expected, η increases significantly with an increase in *α*_*O*_. The η of a single pass was calculated using predicted *d*_32_. The η of single pass was found to be higher and reached up to 0.8 and 2.5% for *α*_*O*_ of 0.05 and 0.15, respectively, due to the small surface area generated by large drops. The surface area increased with the number of passes due to the breakage of large droplets and resulted in a decrease in η (see Figure 10a). The variation in η as a function of *E* may be represented as
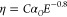
9where *C* is a parameter that varies with the device design and operating conditions. In this study, the value of *C* was found to be 6 for the vortex-based HC device used in this work when operated at 250 kPa pressure drop (see Figure 10b). The value of *C* will be different for different operating pressure and device types. Based on the value of *C*, the performance of different devices can be compared in terms of emulsification efficiency that helps to select the appropriate device for emulsification process. The comparison was made between the η calculated in the present study with literature^11,14,16^ in terms of energy consumption per unit mass of emulsion (*E*) (see Figure 10c). The value of σ was considered 0.012, 0.02 N/m for palm oil–water^40^ and mustard oil–water^41^ systems to calculate η. For comparison, the *α*_*O*_ was scaled down to 0.05 for all the cases.^11,14,16^ The energy consumption of devices^19^ with three slit-cut holes, circular venturi, and slit venturi was significantly higher (>100 kJ/kg) as compared to the vortex-based HC devices (see Figure 10c) to reduce the drop size to ∼0.9 μm. The linear flow devices^19^ showed lower values of η (<0.1%) for producing emulsion with ∼1 μm drop size at *E* of 100–200 kJ/kg. A similar drop size range was generated in the vortex-based HC device with much lower *E* (10–50 kJ/kg). This indicates the vortex-based HC device can be a good alternative over the conventional devices used for emulsification process.

### 4.6 Multiple-Pass CFD-PBM Simulations and Analysis

**Figure 11:**
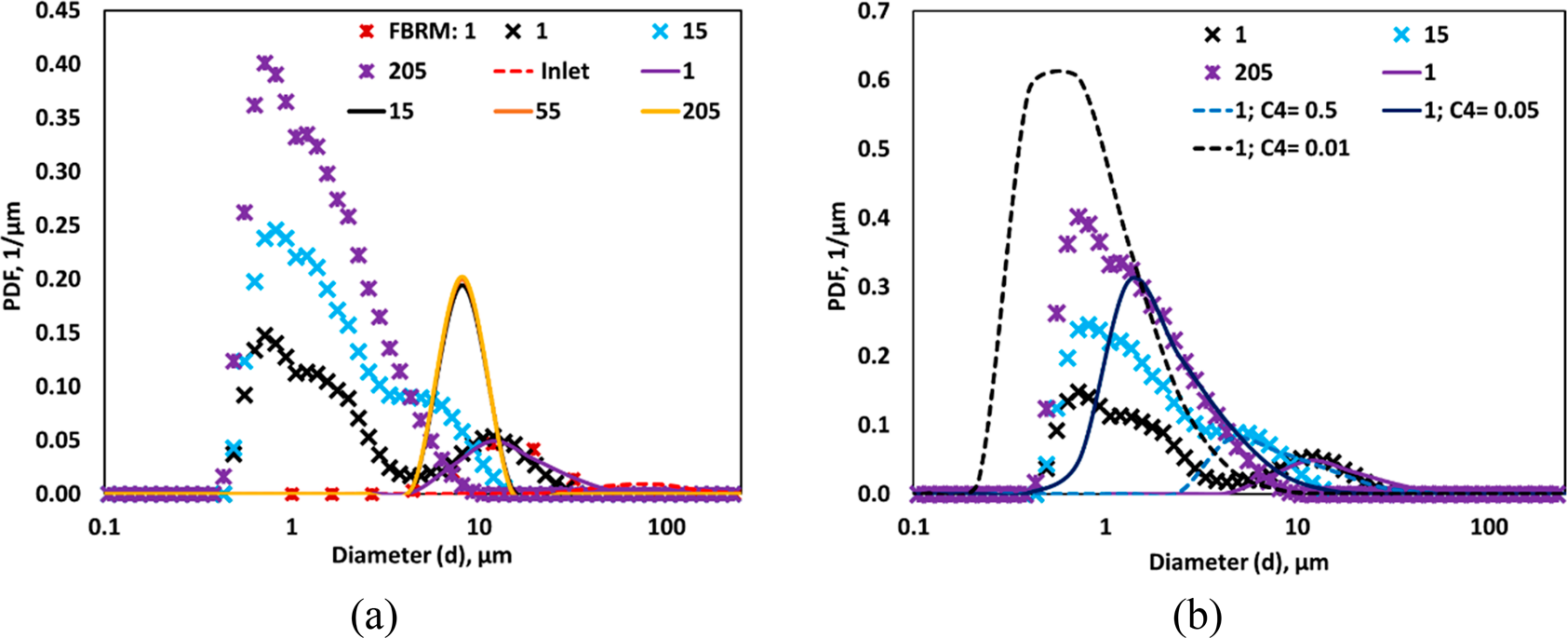
(a) Measured and predicted drop size distribution at a different number of passes. (b) effect of breakage model parameter on drop size distribution (*d*_*T*_ = 3 mm, Δ*P* = 250 kPa, *v*_*t*_ = 2.95 m/s, *α*_*O*_ = 0.05).

The multiple-pass CFD-PBM simulations were performed at Δ*P* = 250 kPa to simulate the flow behavior and drop size distributions. The overall simulated flow behavior in the presence of oil was similar to that reported by Simpson and Ranade^34^ and shown in our previous work.^25^ The PBEs were solved to simulate the DSD and *d*_32_ for multiple passes through the HC device. As mentioned earlier in section 3, the breakage frequency was modeled by the Laakkonen et al.^44^ model to simulate the DSD and *d*_32_ at the outlet for single-pass HC treatment. The expression of breakage frequency *g*(*V*′) is provided in eq (S15) of the Supporting Information. The values of constants *C*_3_, *C*_4_, and *C*_5_ were considered as 650, 1.44, and 0.01 respectively which were found to be suitable for simulating DSD after a single pass through HC simulations.^25^ By considering the same value of constants, the PBM was solved for multiple passes. Figure 11a shows the comparison of simulated and measured DSD for different number of passes. As expected, the second peak of simulated DSD of a single pass was in agreement with the measured DSD. However, the simulated DSD showed a monomodel distribution unlike the bimodel DSD observed in the experiments (with measurements of drop sizes via MS instead of FBRM). The mean drop size of simulated DSD was 13 μm after a single pass and reduced to ∼8 μm after 15 passes, but it was not comparable with the measured DSD. The influence of number of passes was not significant after 15 passes on simulated DSD. This indicates the breakage frequency with the considered value of parameters was not appropriate to capture bimodal nature of DSD and drop breakage below 8 μm.

In our previous study,^25^ we studied the sensitivity of parameters *C*_3_, *C*_4_, and *C*_5_ and found that increase in the value of *C*_3_ was narrowing simulated DSD without significantly affecting the location of peak. On the other hand, *C*_4_ influences both the broadness of simulated DSD as well as the location of the peak. Based on these observations, an attempt was made to adjust values of parameters for realizing better agreement between the simulated and experimental DSD. The value of *C*_4_ was adjusted to obtain the desired location of the peak in the present work (see Figure 11b). The DSD was found to shift toward a lower drop size range with a decrease in *C*_4_. However, the simulated DSD showed significant differences from the measured DSD since the model could not simulate bimodel distribution.

Considering the two distinct mean droplet sizes associated with the bimodal distributions observed in experimentally measured DSD, it appears that the droplet breakage in the vortex-based HC device occurs by two different mechanisms. The first mechanism is related to strongly circulating flow field and associated turbulent energy dissipation rates established in the chamber of vortex diode. The CFD model presented in our earlier work^25^ was shown to be able to capture this mechanism quite well and was able to simulate droplets of the order of 10^1^ μm. The second mechanism might be highly localized intense shear and extremely high turbulent energy dissipation rates generated by collapsing cavities. This second mechanism may be responsible for generating submicrometer-sized droplets (the first peak observed in the bimodal distribution with droplet sizes of the order of 10^0^ μm). The region of collapsing cavities within the HC device is rather small, and all the droplets flowing through the device may not encounter collapsing cavities in a single pass through HC device. The coexistence of these two droplet breakage mechanisms and partial encounter of droplets with collapsing cavities may lead to the observed bimodal distribution (see Figure 3a). In the present CFD-PBM simulations, the DSD generated because of the collapsing cavities (peak 1) was not captured in the simulated results since the localized intense energy dissipation due to collapsing cavities was not included in the model. The simulated DSD of multiple passes therefore showed significant overpredictions and was unable to predict the bimodel nature (see Figure 11a). Therefore, the previous models are limited for predicting the DSD generated through the cavitation devices.

In our previous work,^25^ the liquid–liquid–gas cavitation flow was simulated, and the contours of ε distribution were provided for different operating conditions. Since the high energy dissipation zone was located at the center of the axis in the vortex chamber, the exposure time of drops traveled through those regions was less than that of the low energy dissipation zone in the entire vortex chamber. Therefore, initial droplet breakage may have occurred at the low energy dissipation zone, and the drop size was reduced from ∼66 μm to ∼12 μm after single-pass HC treatment and captured in FBRM measurements^25^ and can also be seen in Figure 2. However, among those droplets, ∼20% of droplets further received exposure to localized intense energy dissipation spots generated due to collapsing cavities. Therefore, the drop size was further reduced from ∼12 μm to ∼5 μm, forming the second peak and resulting in the DSD with a bimodel nature (see Figure 3a) that was missed previously.^25^ As a result of the multiple pass treatment, the exposure time of droplets from travel through the high energy dissipation zone increased and led to a decrease in *d*_32_ from ∼12 μm to ∼1.5 μm after 205 passes (see Figure 3b). The vortex-based HC treatment showed bimodel DSD in the present work in which the first peak represents the size distribution due to the droplet breakage through intense shear by collapsing cavities, and the second peak corresponds to droplet breakage because of the convective flow inside the vortex chamber.

**Figure 12:**
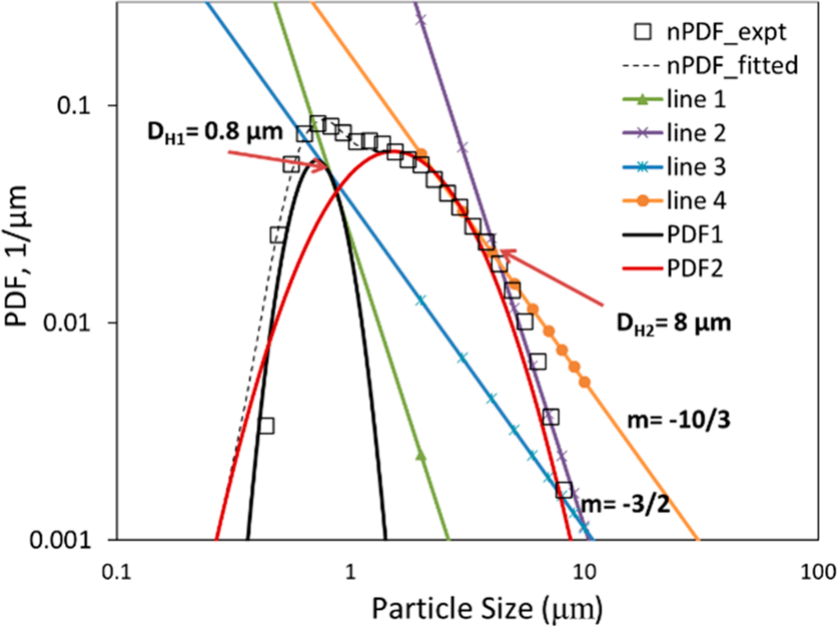
Estimation of Hinze scale diameter (*d*_*H*_) from drop size distribution (*d*_*T*_ = 3 mm, Δ*P* = 250 kPa, *v*_*t*_ = 2.95 m/s, *α*_*O*_ = 0.05).

In the present work, the simulated values of ε inside the vortex chamber were in the range of 1 × 10^5^–2 × 10^5^ m^2^/s^3^. Considering the droplet breakage occurred at the high energy dissipation zone due to the collapsing cavities in the model, the information on ε values in those regions is of utmost importance. This information can be obtained from the Hinze scale analysis of the experimental DSDs. The contribution of different forces responsible for droplet breakage can be determined from the DSD by Hinze scale analysis.^31^ The drop size larger than the Hinze scale diameter shows a −10/3 power-law scaling relationship, and breakage occurs due to the turbulent fragmentation. On the other hand, drop size below the Hinze scale diameter shows a −3/2 power-law scaling relationship mainly due to stabilization caused by the interfacial tension force.^42,43^ The diameter at which change of slope occurs is called as Hinze-scale diameter (*d*_*H*_) which is the drop size in DSD where the both the lines (having −10/3 and −3/2 slops, respectively) intersect with each other^42^ (see Figure 12). The Hinze scale diameter (*d*_*H*_) can be calculated as^44−46^
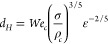
10where *We*_*c*_ is critical weber number, ρ and σ are density of oil and interfacial tension, respectively, and ε is the rate of kinetic energy dissipation. As discussed earlier in this section, the DSD after vortex-based HC treatment showed bimodel distribution. To calculate *d*_*H*_ of each peak, individual distributions of bimodel DSD were determined by applying two log-normal distribution functions with a weightage factor (see Figure 12). Applying the scaling relationships (with −10/3 and −3/2 powers) to both the distributions, the Hinze scales associated with the two peaks (*d*_*H*1_ and *d*_*H*2_) were determined. Several authors reported the order of magnitude of *We*_*c*_ is unity.^47,48^ By taking the value of *We*_*c*_ as unity, eq 10 can be used to estimate a value of ε using the experimentally observed value of Hinze scale diameter. The value of Hinze scale corresponding to the second peak of the bimodal distribution, *d*_*H*2_ was found to be 8 μm (see Figure 12). This value of Hinze scale and eq 10 indicates the value of ε as 1.8 × 10^5^ m^2^/s^3^. This estimated value of ε showed close agreement with values of ε simulated using the CFD model (1 × 10^5^–2 × 10^5^ m^2^/s^3^). This indicates that the second peak generated in the observed bimodel DSD is because of the droplet breakage due to turbulent flow field established in the HC device, and the CFD model presented earlier may be used for simulating the DSD of the second peak.

The Hinze scale diameter determined from the first peak of bimodel distribution, *d*_*H*1_, was found to be 0.8–1 μm (see Figure 12). The magnitude of ε estimated based on this was found to be in the range of 1 × 10^7^–1 × 10^8^ m^2^/s^3^. These values are 2 orders of magnitude larger than the value of ε estimated from the CFD model. Such high values of energy dissipation rates are reported for the collapsing cavities (see the recent study of Pandit et al.).^27^ Collapsing cavities generate highly localized intense energy dissipation rates of similar magnitudes. This suggests that the first peak of the bimodal distribution is generated via droplet breakage caused by collapsing cavities. The CFD-PBM model presented earlier does not explicitly account for such collapsing cavities and therefore is not able to capture generation of bimodal DSD. It is essential to simulate and account for the presence of two different ranges of ε generated by convective flow in the diode chamber and collapsing cavities and their influence of droplet breakage. The cavity dynamics models reported Pandit et al.^27^ need to be integrated with the CFD-PBM models to predict the DSD accurately.

Maindarkar et al.^8^ developed CFD-PBM model to predict bimodel DSD by considering drop breakage under laminar shear and viscous shear force in the colloidal mill. A similar approach may be used to couple droplet breakage by shear generated by convective flow and by intense shear generated by collapsing cavities so that the bimodel DSD generated in vortex-based HC devices may be captured. If direct integration of cavity dynamics models similar to developed by Pandit et al.^27^ with conventional CFD-PBM solvers is difficult, then a surrogate model (that may be based on artificial neural network) representing cavity dynamics may be developed to provide estimates of localized energy dissipation rates generated by collapsing cavities to the conventional CFD-PBM models. This work is in progress and will be published separately. The present work focused on the influence of multiple passes, oil volume fraction, types of oil, and device scale on the drop size distributions. The experimental results and analysis presented in this work provides new and useful information on device-scale performance of vortex-based HC devices for producing liquid–liquid emulsions. The analysis and presented data will be useful for further development of computational models as well as for optimization and scale up of hydrodynamic cavitation devices for producing emulsions.

## 5 Conclusions

Drop breakage in emulsions through multiple pass vortex-based cavitation device was investigated experimentally. The effect of number of passes, liquid–liquid systems, oil volume fraction, and device scales on DSD, *d*_32_, *D*10, *D*50, *D*90, and η was investigated. The multiphase CFD-PBM simulations were performed to predict DSD of multiple passes by considering the breakage parameters and models used in our previous work.^25^ The key findings of the present work are summarized below:

(a) The DSD after vortex-based HC treatment showed bimodel distribution with a smaller peak at ∼10° μm and larger peak at ∼10^1^ μm.

(b) The drop size distribution (DSD) of the RO–water system showed the bimodel nature of DSD. The bimodal nature of DSD was reduced with number of passes and eventually vanished beyond 55 passes. The vortex-based HC device showed excellent drop breakage performance with multiple passes and was able to reduce Sauter mean diameter *d*_32_ for RO in water emulsions from 66 μm to ∼2 μm after 35 passes and ∼1 μm beyond 105 passes. The *d*_32_ (in micrometers) was found to vary with energy dissipation rate per unit mass (ε̅, *m*^2^/*s*^3^) and number of passes (*n*_*P*_) and overall relationship is reasonably represented by eq 2.

(c) Monomodel DSD was observed for the TCE–water system. The *d*_32_ was significantly lower in TCE–water system at 15 passes (1 μm) than the RO–water system (2.5 μm).

(d) The influence of oil volume fraction (*α*_*O*_) on DSDs, diameters, and η was investigated, and marginal differences were found in *d*_32_ between *α*_*O*_ of 0.05 and 0.15.

(e) The DSD of the bench-scale device (*d*_*T*_ = 12 mm; *Q* = 20 LPM) showed larger drop sizes compared to the lab-scale device (*d*_*T*_ = 3 mm; *Q* = 1 LPM). The drop size obtained after 15 passes (*D*90 = 9.78 μm; *d*_32_ = 2.3 μm) in the lab-scale device was found in the bench-scale device after nearly 55 passes (*D*90= 10 μm; *d*_32_ = 2.3 μm). For the same *E* (*E* = 9 kJ/kg), the *d*_32_ of bench-scale and lab-scale device was found to be 2.5 and 2 μm, respectively.

(f) The breakage efficiency (η) of the vortex-based HC device was calculated and compared against energy consumption (*E*). The η of the vortex-based device was found to be directly proportional to α_O_ and *E*^–0.8^ (see eq 9). The η of vortex-based HC device approached 2.5% at *α*_*O*_ of 0.15 for a single pass. The η of vortex-based HC device showed almost 10 times higher values at low (*E*) as compared to a high-pressure homogenizer and other cavitation devices (orifice and venturi).

(g) The CFD-PBM model developed in the previous work^25^ was unable to capture the bimodal nature of DSDs. The most likely reason is the absence of appropriate representation of highly localized intense energy dissipation generated by collapsing cavities. The order of magnitude of localized energy dissipation rates for collapsing cavities was estimated using Hinze scale analysis. These estimates showed good agreement with the energy dissipation rates reported for collapsing cavities.

The presented experimental results, analysis, and developed correlations will be useful for developing better multiphase CFD-PBM models and for harnessing hydrodynamic cavitation for producing fine emulsions.

## Supporting Information Available

Photographs of typical experimental setups of vortex-based hydrodynamic cavitation, model equations and geometric information on CFD simulations, sensitivity of bins in PBM model and information on *D*10, *D*50 and *D*90 for different Δ*P* (PDF)

## Nomenclature

*A*_*i*_: area of cell *i*, m^2^
*A*_*net*_: rate of net surface area generation, m^2^/s
*C*: constant for breakage efficiency (eq 9)
*d*_*min*_: minimum stable diameter, μm
*d*_32_: Sauter mean diameter, μm
*d*_321_: Sauter mean diameter of single pass treatment, μm
*d*_*H*_: Hinze scale diameter, μm
*d*_*T*_: throat diameter, mm
*E*: energy consumption per unit mass of emulsion, J/kg
*Eu*: Euler number, -
*g*(*V*′): breakage frequency, 1/s
*n*_*p*_: number of passes, -
Δ*P*: inlet pressure drop, kPa
*Q*: volumetric flow rate, LPH
*t*: time, s
*v*_*i*_: velocity of cell *i*, m/s
*v*_*t*_: throat velocity, m/s
*We*_*c*_: critical Weber number, -
**Greek Letters**
α: volume fraction, -
ρ: density, kg/m^3^
μ: viscosity, kg/ms
σ: interfacial tension, N/m
η: energy efficiency for droplet breakage, %
ε̅: average energy dissipation rate per unit mass in the device, m^2^/s^3^
**Subscript**
O: organic phase
A: aqueous phase
**Acronyms**
CFD: computational fluid dynamics
CLD: chord length distribution
DSD: drop size distribution
FBRM: focused beam reflectance measurements
HC: hydrodynamic cavitation
PBE: population balance equation
PBM: population balance model
PDF: probability density function
RO: rapeseed oil
TCE: trichloroethylene

## References

[ref1] Gupta A.; Eral H. B.; Hatton T. A.; Doyle P. S. Nanoemulsions: formation, properties and applications. Soft Matter 2016, 12 (11), 2826–2841. 10.1039/C5SM02958A. 26924445

[ref2] Groeneweg F.; van Dieren F.; Agterof W. G. M. Droplet break-up in a stirred water-in-oil emulsion in the presence of emulsifiers. Colloids Surf., A 1994, 91, 207–214. 10.1016/0927-7757(94)02913-X.

[ref3] Becker P. J.; Puel F.; Chevalier Y.; Sheibat-Othman N. Monitoring silicone oil droplets during emulsification in stirred vessel: Effect of dispersed phase concentration and viscosity. Can. J. Chem. Eng. 2014, 92 (2), 296–306. 10.1002/cjce.21885.

[ref4] Khalil A.; Puel F.; Chevalier Y.; Galvan J.-M.; Rivoire A.; Klein J.-P. Study of droplet size distribution during an emulsification process using in situ video probe coupled with an automatic image analysis. Chem. Eng. J. 2010, 165 (3), 946–957. 10.1016/j.cej.2010.10.031.

[ref5] Wang X.; Jiang Y.; Wang Y. W.; Huang M. T.; Ho C. T.; Huang Q. Enhancing anti-inflammation activity of curcumin through O/W nanoemulsions. Food Chem. 2008, 108 (2), 419–424. 10.1016/j.foodchem.2007.10.086. 26059118

[ref6] Li Y.; Xiang D. Stability of oil-in-water emulsions performed by ultrasound power or high-pressure homogenization. PLoS One. 2019, 14 (3), e0213189 10.1371/journal.pone.0213189. 30849091 PMC6407764

[ref7] Maindarkar S.; Dubbelboer A.; Meuldijk J.; Hoogland H.; Henson M. Prediction of emulsion drop size distributions in colloid mills. Chem. Eng. Sci. 2014, 118, 114–125. 10.1016/j.ces.2014.07.032.

[ref8] Perrier-Cornet J. M.; Marie P.; Gervais P. Comparison of emulsification efficiency of protein-stabilized oil-in-water emulsions using jet, high pressure and colloid mill homogenization. J. Food Eng. 2005, 66 (2), 211–217. 10.1016/j.jfoodeng.2004.03.008.

[ref9] O’Sullivan J.; Murray B.; Flynn C.; Norton I. Comparison of batch and continuous ultrasonic emulsification processes. J. Food Eng. 2015, 167, 114–121. 10.1016/j.jfoodeng.2015.05.001.

[ref10] Servant G.; Laborde J. L.; Hita A.; Caltagirone J. P.; Gerard A. Spatio-temporal dynamics of cavitation bubble clouds in a low frequency reactor: comparison between theoretical and experimental results. Ultrason. Sonochem. 2001, 8 (3), 163–174. 10.1016/S1350-4177(01)00074-8. 11441594

[ref11] Parthasarathy S.; Siah Ying T.; Manickam S. Generation and Optimization of Palm Oil-Based Oil-in-Water (O/W) Submicron-Emulsions and Encapsulation of Curcumin Using a Liquid Whistle Hydrodynamic Cavitation Reactor (LWHCR). Ind. Eng. Chem. Res. 2013, 52 (34), 11829–11837. 10.1021/ie4008858.

[ref12] Tang S. Y.; Shridharan P.; Sivakumar M. Impact of process parameters in the generation of novel aspirin nanoemulsions--comparative studies between ultrasound cavitation and microfluidizer. Ultrason. Sonochem. 2013, 20 (1), 485–497. 10.1016/j.ultsonch.2012.04.005. 22633626

[ref13] Ashokkumar M.; Rink R.; Shestakov S.; Tee K. Hydrodynamic cavitation - an alternative to ultrasonic food processing. Tech. Acoustics 2011, 9, 1–10.

[ref14] Ramisetty K. A.; Pandit A. B.; Gogate P. R. Novel Approach of Producing Oil in Water Emulsion Using Hydrodynamic Cavitation Reactor. Ind. Eng. Chem. Res. 2014, 53 (42), 16508–16515. 10.1021/ie502753d.

[ref15] Zhang Z.; Wang G.; Nie Y.; Ji J. Hydrodynamic cavitation as an efficient method for the formation of sub-100 nm O/W emulsions with high stability. Chin. J. Chem. Eng. 2016, 24 (10), 1477–1480. 10.1016/j.cjche.2016.04.011.

[ref16] Carpenter J.; George S.; Saharan V. K. Low pressure hydrodynamic cavitating device for producing highly stable oil in water emulsion: Effect of geometry and cavitation number. Chem. Eng. Process. 2017, 116, 97–104. 10.1016/j.cep.2017.02.013.

[ref17] Fesenko A.; Yevsiukova F.; Basova Y.; Ivanova M.; Ivanov V. Prospects of Using Hydrodynamic Cavitation for Enhancement of Efficiency of Fluid Working Medium Preparation Technologies. Period. Polytech. Mech. Eng. 2018, 62 (4), 269–276. 10.3311/PPme.11877.

[ref18] Urban K.; Wagner G.; Schaffner D.; Röglin D.; Ulrich J. Rotor-Stator and Disc Systems for Emulsification Processes. Chem. Eng. Technol. 2006, 29 (1), 24–31. 10.1002/ceat.200500304.

[ref19] Sarvothaman V. P.; Simpson A. T.; Ranade V. V. Modelling of vortex based hydrodynamic cavitation reactors. Chemical Engineering Journal. 2019, 377, 119639 10.1016/j.cej.2018.08.025.

[ref20] Ramisetty K. A.; Pandit A. B.; Gogate P. R. Ultrasound assisted preparation of emulsion of coconut oil in water: Understanding the effect of operating parameters and comparison of reactor designs. Chem. Eng. Process. 2015, 88, 70–77. 10.1016/j.cep.2014.12.006.

[ref21] Ji J.; Wang J.; Li Y.; Yu Y.; Xu Z. Preparation of biodiesel with the help of ultrasonic and hydrodynamic cavitation. Ultrasonics. 2006, 44, e411–e414. 10.1016/j.ultras.2006.05.020. 16797656

[ref22] Soldo. Emulsification with Soldo Cavitators Technology, 2022. https://soldocavitators.com/omogeneizzazione-industriale/#emulsificazione. (Accessed 02/08/2022).

[ref23] Kozyuk O. V. Use of hydrodynamic cavitation for emulsifying and homogenizing processes. Am. Lab. 1999, 31, 6–8.

[ref24] Simpson A.; Ranade V. V. 110th Anniversary: Comparison of Cavitation Devices Based on Linear and Swirling Flows: Hydrodynamic Characteristics. Ind. Eng. Chem. Res. 2019, 58 (31), 14488–14509. 10.1021/acs.iecr.9b02757.

[ref25] Thaker A. H.; Ranade V. V. Drop breakage in a single-pass through vortex-based cavitation device: Experiments and modeling. AIChE J. 2021, e17512 10.1002/aic.17512.

[ref26] Thaker A. H.; Ranade V. V. Towards harnessing hydrodynamic cavitation for producing emulsions: Breakage of an oil drop in a vortex based cavitation device. Chem. Eng. Process. 2022, 180, 108753 10.1016/j.cep.2021.108753.

[ref27] Pandit A. V.; Sarvothaman V. P.; Ranade V. V. Estimation of chemical and physical effects of cavitation by analysis of cavitating single bubble dynamics. Ultrason. Sonochem. 2021, 77, 105677 10.1016/j.ultsonch.2021.105677. 34332329 PMC8339230

[ref28] Ranade V. V., Kulkarni A. A., Bhandari V. M. Vortex diodes as effluent treatment devices. U.S. Patent No. 9,422,952B422, 2016.

[ref29] Simpson A.; Ranade V. V. Flow characteristics of vortex based cavitation devices. AIChE J. 2019, 65 (9), e16675 10.1002/aic.16675.

[ref30] Ranade N. V.; Sarvothaman V.; Ranade V. V. Acoustic Analysis of Vortex-based Cavitation Devices: Inception and extent of cavitation. Ind. Eng. Chem. Res. 2021, 60 (22), 8255–8268. 10.1021/acs.iecr.1c01005.

[ref31] Chatzi E.; Kiparissides C. Dynamic simulation of bimodal drop size distributions in low-coalescence batch dispersion systems. Chem. Eng. Sci. 1992, 47 (2), 445–456. 10.1016/0009-2509(92)80032-8.

[ref32] Janssen J. M. H.; Meijer H. E. H. Droplet breakup mechanisms: Stepwise equilibrium versus transient dispersion. J. Rheol. 1993, 37 (4), 597–608. 10.1122/1.550385.

[ref33] Carpenter J.; Pinjari D. V.; Kumar Saharan V.; Pandit A. B. Critical Review on Hydrodynamic Cavitation as an Intensifying Homogenizing Technique for Oil-in-Water Emulsification: Theoretical Insight, Current Status, and Future Perspectives. Ind. Eng. Chem. Res. 2022, 61 (30), 10587–10602. 10.1021/acs.iecr.2c00754.

[ref34] Rayner M. Scales and Forces in Emulsification. Contemp. Food Eng. 2015, 3–32. 10.1201/b18436-3.

[ref35] Håkansson A. A hydrodynamic comparisons of two different high-pressure homogenizer valve design principles: A step towards increased efficiency. Chem. Eng. Res. Des. 2022, 184, 303–314. 10.1016/j.cherd.2022.06.009.

[ref36] Hofmann M.; Bayles A. V.; Vermant J. Stretch, fold, and break: Intensification of emulsification of high viscosity ratio systems by fractal mixers. AIChE J. 2021, 67 (5), e17192 10.1002/aic.17192.

[ref37] Di Giuliano A.; Funcia I.; Pérez-Vega R.; Gil J.; Gallucci K. Novel Application of Pretreatment and Diagnostic Method Using Dynamic Pressure Fluctuations to Resolve and Detect Issues Related to Biogenic Residue Ash in Chemical Looping Gasification. Processes 2020, 8 (9), 1137 10.3390/pr8091137.

[ref38] Yoon H.; Oostrom M.; Werth C. J. Estimation of interfacial tension between organic liquid mixtures and water. Environ. Sci. Technol. 2009, 43 (20), 7754–7761. 10.1021/es901061k. 19921890

[ref39] Gaikwad S. G.; Pandit A. B. Ultrasound emulsification: effect of ultrasonic and physicochemical properties on dispersed phase volume and droplet size. Ultrason. Sonochem. 2008, 15 (4), 554–563. 10.1016/j.ultsonch.2007.06.011. 17698396

[ref40] Ho C. C.; Chow M. C. The effect of the refining process on the interfacial properties of palm oil. J. Amer. Oil Chem. Soc. 2000, 77 (2), 191–199. 10.1007/s11746-000-0031-7.

[ref41] Ghosh V.; Mukherjee A.; Chandrasekaran N. Mustard oil microemulsion formulation and evaluation of bactericidal activity. Int. J. Pharm. Pharm. Sci. 2012, 4 (4), 497–500.

[ref42] Wang Z.; Yang J.; Stern F. High-fidelity simulations of bubble, droplet and spray formation in breaking waves. J. Fluid Mech. 2016, 792, 307–327. 10.1017/jfm.2016.87.

[ref43] Rivière A.; Mostert W.; Perrard S.; Deike L. Sub-Hinze scale bubble production in turbulent bubble break-up. J. Fluid Mech. 2021, 917, A40 10.1017/jfm.2021.243.

[ref44] Hinze J. O. Fundamentals of the hydrodynamic mechanism of splitting in dispersion processes. AIChE J. 1955, 1 (3), 289–295. 10.1002/aic.690010303.

[ref45] Deane G. B.; Stokes M. D. Scale dependence of bubble creation mechanisms in breaking waves. Nature 2002, 418 (6900), 839–844. 10.1038/nature00967. 12192401

[ref46] Gupta A.; Eral H. B.; Hatton T. A.; Doyle P. S. Controlling and predicting droplet size of nanoemulsions: scaling relations with experimental validation. Soft Matter 2016, 12 (5), 1452–1458. 10.1039/C5SM02051D. 26646895

[ref47] Jain S. S. Flow-induced breakup of drops and bubbles. arXiv (Fluid Dynamics), Jan. 22, 2017, arXiv:1701.06157, ver. 1. https://doi.org/10.48550/arXiv.1701.06157 (Accessed on 03/08/2022).

[ref48] Saha A.; Lee J. D.; Basu S.; Kumar R. Breakup and coalescence characteristics of a hollow cone swirling spray. Phys. Fluids 2012, 24 (12), 124103 10.1063/1.4773065.

