# Supplementary material for: Emulsions Using a Vortex-Based Cavitation Device: Influence of Number of Passes, Pressure Drop, and Device Scale on Droplet Size Distributions
Source: Ind Eng Chem Res. 2022 Dec 19;62(45):18837–51. doi: 10.1021/acs.iecr.2c03714 (PMC10655102; doi:10.1021/acs.iecr.2c03714)
Supplement: Supplementary file 1 — ie2c03714_si_001.pdf [file ie2c03714_si_001.pdf]

Supplementary Information

Emulsions using Vortex based Cavitation Device:
Influence of number of passes, pressure drop and device scale on droplet size distributions

Abhijeet H. Thaker and Vivek V. Ranade*
Multiphase Reactors and Intensification Group
Bernal Institute, University of Limerick, Limerick V94T9PX, Ireland
*Email: vivek.ranade@ul.ie

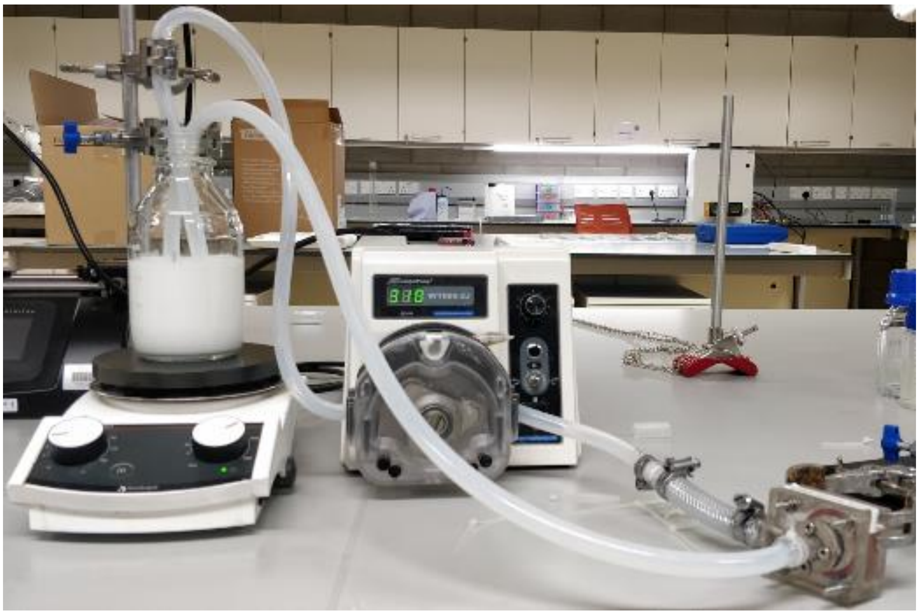

Figure S1: Photograph of lab-scale experimental set-up ($d_t = 3\ mm$).

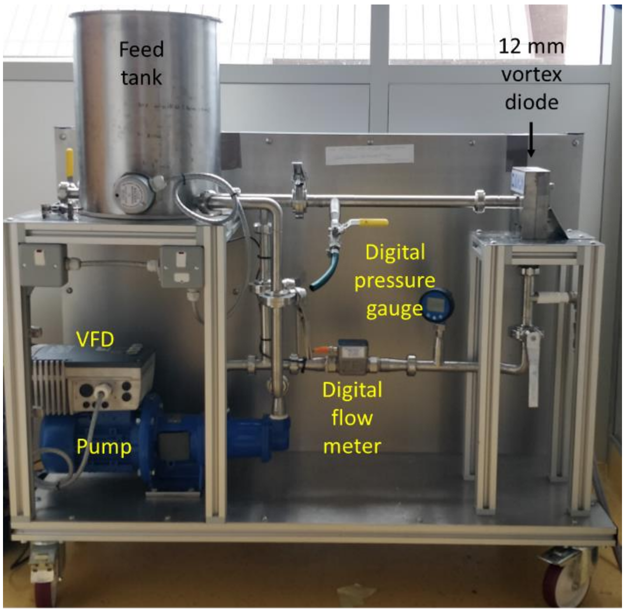

Figure S2: Photograph of bench-scale experimental set-up ($d_t = 12\ mm$).

1. Multiphase *CFD* model

In the present study, the multiphase flow was considered for three different phases, i.e., water (primary), water vapour (through cavitation) and oil (secondary). The following governing equations based on the mixture model were solved[1]:

$$\frac{\partial \rho_m}{\partial t} + \nabla \cdot \rho_m \vec{v}_m = 0 \qquad \text{(S1)}$$

where, $\vec{v}_m$ is the mass-averaged velocity and defined as

$$\vec{v}_m = \frac{\sum_{k=1}^{q} \alpha_k \rho_k \vec{v}_k}{\rho_m} \qquad \text{(S2)}$$

where, mixture density, ρ_m defined as $\rho_m = \sum_{k=1}^{q} \alpha_k \rho_k$, where, α_k is the volume fraction of phase k and q is total number of phases.

$$\frac{\partial \rho_m \vec{v}_m}{\partial t} + \nabla \cdot \rho_m \vec{v}_m \vec{v}_m = -\nabla P + \nabla \cdot [\mu_m (\nabla \vec{v}_m + \vec{v}_m^T)] + \rho_m g + F - \nabla \cdot \left[\sum_{k=1}^{q} \alpha_k \rho_k \vec{v}_{dr,k} \vec{v}_{dr,k}\right] \qquad \text{(S3)}$$

where, μ_m is viscosity of the mixture. F represents external body force. In the present simulation, only drag force that arise from interaction of aqueous (primary) phase with oil or vapour phase, was considered as a body force.

$$\mu_m = \sum_{k=1}^{q} \alpha_k \mu_k \qquad \text{(S4)}$$

The slip velocity $\vec{v}_{kA}$ is defined as the velocity of secondary phases, k (oil or vapour phases) relative to the velocity of the primary phase A:

$$\vec{v}_{kA} = \vec{v}_k - \vec{v}_A \qquad \text{(S5)}$$

The drift velocity $\vec{v}_{dr}$ and slip velocity are connected $\vec{v}_{kA}$ as[1]:

$$\vec{v}_{dr,k} = \vec{v}_{kA} - \sum_{k=1}^{q} c_k \vec{v}_{kA} \qquad \text{(S6)}$$

wherer c_k is mass fraction of any phase (k) is defined as $c_k = \frac{\alpha_k \rho_k}{\rho_m}$

$$\vec{v}_{kA} = \frac{24}{Re} \frac{(\rho_k - \rho_m) d_k^2}{18 \mu_A C_D} \left(g - \frac{D v_m}{Dt}\right) \qquad \text{(S7)}$$

The drag coefficient C_D is defined as following[2]:

$$C_D = \begin{cases} \frac{24}{Re}(1 + 0.15 Re^{0.687}) & Re \leqslant 1000 \\ 0.44 & Re > 1000 \end{cases} \qquad \text{where, } Re = \frac{\rho_m \vec{v}_{kA} d_k}{\mu_A} \qquad \text{(S8)}$$

Eq. S8 was used to estimate drag coefficient between dispersed oil drop and water as well as dispersed gas/vapour bubble and water with appropriate dispersed phase diameter and Reynolds number.

Based on the recommendations of Simpson and Ranade[3], *SST k-ω* model was used to simulate turbulent flow. The turbulence kinetic energy and specific dissipation rate were calculated using the following equations[1]:

$$\frac{\partial}{\partial t}(\rho_m k) + \frac{\partial}{\partial_x}(\rho_m k \vec{v}_m) = \frac{\partial}{\partial x_j}\left(\Gamma_k \frac{\partial k}{\partial_x}\right) + G_k - Y_k \quad \text{(S9)}$$

$$\frac{\partial}{\partial t}(\rho_m \omega) + \frac{\partial}{\partial_x}(\rho_m \omega \vec{v}_m) = \frac{\partial}{\partial x_j}\left(\Gamma_\omega \frac{\partial \omega}{\partial_x}\right) + G_\omega - Y_\omega \quad \text{(S10)}$$

where G_k and G_ω are the generation of turbulence kinetic energy k and ω, respectively, due to the mean velocity gradients. Y_k and Y_ω indicate the dissipation of k and ω due to turbulence. Since the Eulerian Mixture model was used in the present work, the turbulence equations were solved for mixture and not per phase. The generation and dissipation source terms reported by Simpson and Ranade[3] were used in this work.

Several models have been proposed for simulating cavitation e.g., Zwart et al.[4], Schnerr and Sauer[5] and Singhal et al.[6]. The Singhal et al.[6] model was used in this work since it considers the term of non-condensable gas, which is relevant in the present system. The Singhal et al.[6] cavitation model equations are:

$$\frac{\partial}{\partial t}(\rho_\mathrm{m} f) + \nabla \cdot (\rho_\mathrm{m} \vec{v}_\mathrm{m} f) = \nabla \cdot (\Gamma \nabla f) + R_\mathrm{e} - R_\mathrm{c} \quad \text{(S11)}$$

f is vapour mass fraction and Γ effective diffusion coefficient. R_e and R_c are the mass source and sink terms for the evaporation and condensation, respectively. The R_e and R_c are formulated as:

$$R_e = C_1 \frac{\sqrt{k}}{\sigma} \rho_v \rho_A \left[\frac{2}{3}\left(\frac{P_v - P}{\rho_A}\right)\right]^{1/2} \left(1 - f_\mathrm{v} - f_\mathrm{g}\right) \quad \text{(S12)}$$

$$R_\mathrm{c} = C_2 \frac{\sqrt{k}}{\sigma} \rho_\mathrm{v} \rho_A \left[\frac{2}{3}\left(\frac{P - P_v}{\rho_A}\right)\right]^{1/2} f_\mathrm{v} \quad \text{(S13)}$$

where ρ_v is density of the vapour. f_g represents the mass fraction of non-condensable gas. The values of empirical constants C_1 and C_2 were considered 0.02 and 0.01, respectively, as recommended in the original article[6].

1.1. Population balance model

Drop breakage and evolution of drop size distribution was modelled using the following population balance equation *(PBM)*. With the surfactant concentration used in this work, the emulsions and *DSDs* were found to be stable over days (see Figure 3b of the manuscript). Thus, the coalescence time scale was several orders of magnitude larger than the residence time of the vortex chamber (~ 0.1 s). The coalescence was therefore not considered in the present work. Previous studies of Konno et al.[7] and Maaβ et al.[8,9] also support the assumption of negligible coalescence in the systems similar to investigated in this work. The *PBM* for pure breakage system can be written as[1]:

$$\frac{\partial n(V,t)}{\partial t} + \nabla \cdot \left(\vec{v}_{\mathrm{m}} n(V,t)\right) = \int_{v}^{\infty} p g(V') \beta(V/V') n\,(V',t) dV' - g(V) n(V,t) \qquad \text{(S14)}$$

where, $n(V,t)dV$ is the number density of drops with volume in the range of volume V to $V + dV$ at time t. The first term on the right-hand side represents the birth of drops with volume V due to the breakage of parent drops of volume V'. The second terms represents the loss of drops with volume V due to the breakage. $g(V')$ is breakage frequency that represents the number of parent drops with volume V' breaking per unit time. $p(V')$ is the number of daughter droplets generated by breakage of parent drop with volume V'. In the present model, the value of p was considered 2. $\beta(V/V')$ is the daughter droplet distribution function that defines the probability of generating daughter drop of volume V from the breakage of parent drop with volume V'.

The Laakkonen et al.[10] model which considers both the surface and viscous forces that relevant in the present work was used for breakage frequency $g(V')$. The expression of $g(V')$ considered from Laakkonen et al.[10] is:

$$g(V') = C_3 \varepsilon^{1/3} \mathrm{erfc}\left(\sqrt{C_4 \frac{\sigma}{\rho_A \varepsilon^{2/3} d^{5/3}} + C_5 \frac{\mu_O}{\sqrt{\rho_A \rho_O}\, \varepsilon^{1/3} d^{4/3}}}\right) \qquad \text{(S15)}$$

d is drop diameter and μ_O is viscosity of the oil phase. C_3, C_4 and C_5 are model parameters. Eq. S15 contains two terms: the first term containing C_3 represents the frequency of the droplet breakage, and the second term containing C_4 represents the probability of breakage given in terms of the complementary error function (erfc)[11]. The parameter C_3 influences breakage frequency and thereby influences the broadness of *DSD* (without significantly affecting the location of the peak in *DSD*). The parameter C_4 influences the breakage probability and thereby influences both the broadness as well as peak location of *DSD*. Therefore, the value of C_4 was adapted in the present work. The daughter droplet distribution function [$\beta(V/V')$] was considered to be a parabolic probability distribution function and is represented as:

$$\beta(V/V') = 0.5\left[\frac{C}{V'} + \frac{1 - C/2}{V'}\left\{24\left(\frac{V}{V'}\right)^2 - 24\left(\frac{V}{V'}\right) + 6\right\}\right] \qquad \text{(S16)}$$

where, C= 1.

The evolution of drop population (*DSD*) was tracked using the discrete method (method of class)[12]. This method represents continuous *DSD* in terms of discrete size groups. The population balance equation was reformulated in terms of the volume fraction of drop size i and represented as[1]:

$$\frac{\partial \rho_m \alpha_{O,i}}{\partial t} + \nabla \cdot \left(\rho_m \alpha_{O,i} \vec{v}_{\mathrm{m}}\right) = \rho_O V_i (B_i - D_i) \qquad \text{(S17)}$$

where $\alpha_{O,i}$ is the oil volume fraction of representative group of drop size i. B_i and D_i are rates of birth and loss of drops of size i due to the breakage per unit volume, respectively.

$$\alpha_{O,i} = N_i V_i \qquad \text{(S18)}$$

where N_i is total number of drops of size i per unit volume and calculated as:

$$N_i(t) = \int_{V_i}^{V_{i+1}} n(V,t) dV \qquad \text{(S19)}$$

where, V_i is volume of representative group for drop size i.

The oil volume fraction of all the groups (α_O) is defined as:

$$\alpha_O = \sum_{i=1}^{M} \alpha_{O,i} \qquad \text{(S20)}$$

The birth and loss terms were discretised as:

$$B_i = \sum_{j=i+1}^{M} p_j g(V_j) N_j \beta(V_i/V_j) \text{ and } D_i = g(V_i) N_i \qquad \text{(S21)}$$

The Sauter mean diameter (d_{32}) was calculated based on the organic phase volume fraction as:

$$d_{32} = \sum_{i=1}^{M} d_i^3 \alpha_{O,i} / \sum_{i=1}^{M} d_i^2 \alpha_{O,i} \qquad \text{(S22)}$$

The probability density function *(PDF)* was calculated by dividing $\alpha_{O,i}$ with the width of group i (Δx_i) as ($PDF = \alpha_{O,i}/\Delta x_i$).

1.2. Solution domain, boundary conditions

The outline of computational geometry, solution domain with the location of inlet and outlet is shown in Figure S3. The simulations were performed for a small scale *HC* device (d_T= 3 mm). The axial port length of the HC device was extended up to $33d_T$ to avoid the backflow at the outlet during the simulation in the present work. The total length of the extended axial port was therefore set to be 133 mm. The other geometrical dimensions of *HC* device were kept the same as considered in our previous work[1]. The velocity inlet boundary condition was considered for both the phases (water and oil) at the inlet of the *HC* device. The throat velocity (v_t) was set as 2.96 m/s corresponding ΔP of 250 kPa. The emulsion was freely discharged to the holding tank before the recirculation. Therefore, a pressure outlet

boundary condition with fixed pressure of 1 atm was used at the outlet. The no-slip wall condition was considered at all the surfaces of *HC* device.

The CFD- PBM model equations were solved using commercial solver *ANSYS Fluent 2020R1*. The breakage frequency in *PBEs* was modelled using Laakkonen et al.[2] model. The value of model parameter, C_4 in Laakkonen et al.[2] model (see Eq. 15 of the Supplementary Information) was modified using a user-defined function *(UDF)* in *Fluent*. The sensitivity of computational mesh and time-step of transient simulations was performed in our previous work[3] and identified that the results were not sensitive after 2 million of cells and time step of 10^{-4} s. The SIMPLE algorithm was used for pressure velocity coupling and the PRESTO! discretisation scheme considered for pressure equations. A second-order discretisation scheme was employed for momentum and turbulence quantities. A convergence criterion of 10^{-6} and 10^{-8} was used for flow equations and for *PBEs*, respectively. The *Fluent 2020 R1* meshing module[13] was used to generate Mosaic (structured hexahedral dominant) mesh which found adequate for vortex diode geometry. Detail information on mesh quality and sensitivity is provided in previous work[3].

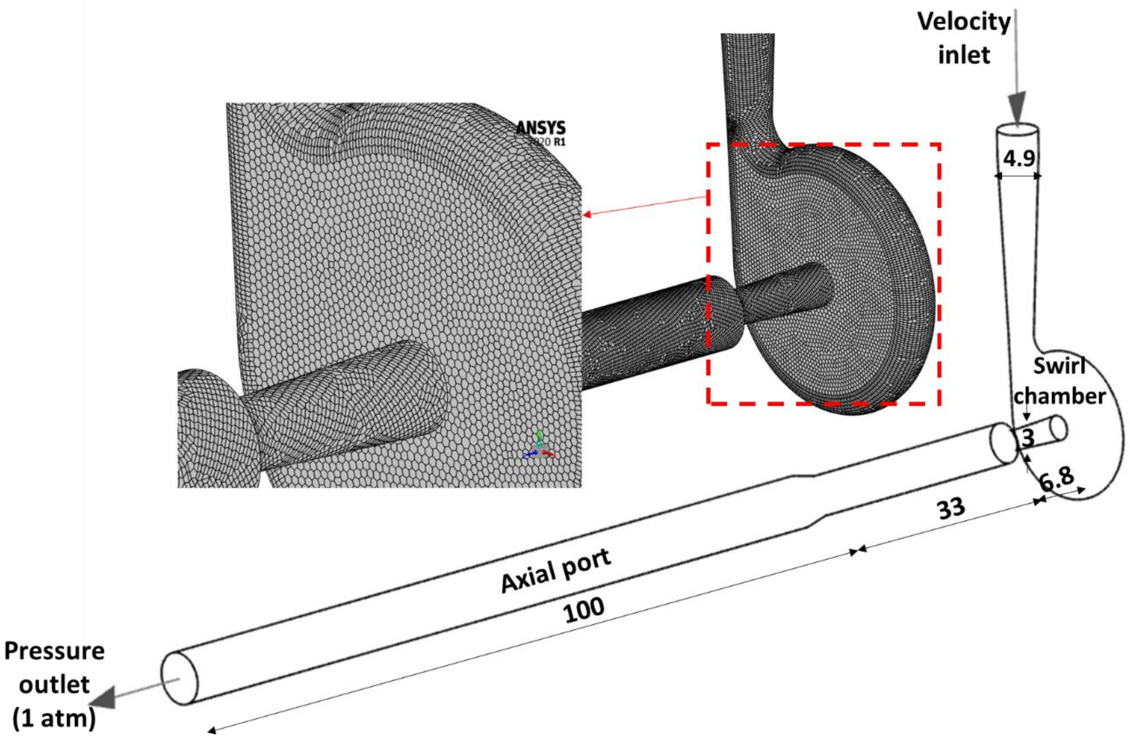

Figure S3: (a) Solution domain, computational geometry and boundary conditions and (b) computational grid of vortex diode (all dimensions are in 'mm')

The method of classes was used for solving PBEs. we used 20 unequal sized bins for simulating overall *DSD*. As mentioned earlier, the volume fraction of representative drop size group is defined as $\alpha_{o,i}$ where $i = 1\ to\ 20$, therefore, $\sum_{i=1}^{20} \alpha_{o,i} = \alpha_o$.

While solving equations using *ANSYS Fluent*, $\alpha_{O,i}$ was determined by introducing a new solution variable γ_i as[1]:

$$\gamma_i = \frac{\alpha_{o,i}}{\alpha_o} \text{ where } \sum_{i=1}^{M} \gamma_i = 1 \quad \text{(S23)}$$

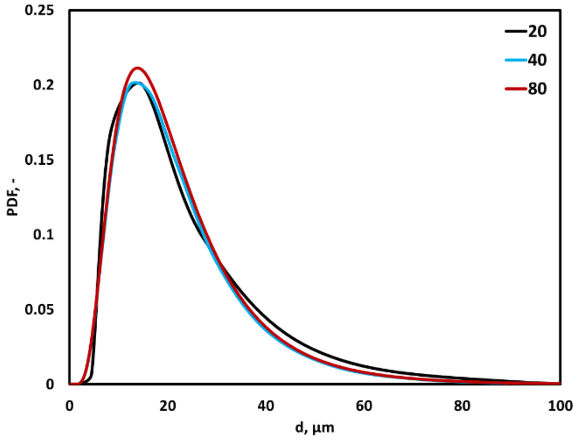

Figure S4: Effect of number of bins on the drop size distribution

Table S1: $D10$, $D50$ and $D90$ for different ΔP

ΔP, kPa	$D10$	$D50$	$D90$
**50**	2.6	22.0	39.1
	2.0	17.6	31.0
	1.9	16.7	29.1
	1.7	14.5	25.4
	1.7	14.0	24.6
**150**	1.2	8.5	17.9
	1.1	7.4	15.5
	1.1	6.9	14.6
	1.1	6.6	13.9
	1.0	6.0	12.8
**250**	1.0	3.9	9.2
	0.9	2.8	7.4
	0.8	2.7	7.0
	0.8	2.2	5.6
	0.7	2.0	5.0

References

1. Ansys® Academic Research Mechanical and CFD, Release 2020 R1, Help System, Fluent Theory Guide, ANSYS, Inc.
2. Schiller, L. & Naumann, Z. A Drag Coefficient Correlation. *Z. Ver. Deutsch. Ing* **77**, (1935).
3. Simpson, A. & Ranade, V. V. Flow characteristics of vortex based cavitation devices: Computational investigation on influence of operating parameters and scale. *AIChE Journal* **65**, 1–18 (2019).
4. Zwart, P., Belamri, T. & Technology, A. S. A two-phase flow model for predicting cavitation dynamics. (2019).
5. Sauer, J. & Schnerr, G. H. Unsteady Cavitating Flow: A New Cavitation Model Based on Modified Front Capturing Method and Bubble Dynamics. (2016).
6. Singhal, A. K., Athavale, M. M., Li, H. & Jiang, Y. Mathematical Basis and Validation of the Full Cavitation Model. *Journal of Fluids Engineering* **124**, 617 (2002).
7. Konno, M., Aoki, M. & Saito, S. Scale effect on breakup process in liquid-liquid agitated tanks. *Journal of Chemical Engineering of Japan* **16**, 312–319 (1983).
8. Maaß, S. & Kraume, M. Determination of breakage rates using single drop experiments. *Chemical Engineering Science* **70**, 146–164 (2012).
9. Maaß, S., Wollny, S., Sperling, R. & Kraume, M. Numerical and experimental analysis of particle strain and breakage in turbulent dispersions. *Chemical Engineering Research and Design* **87**, 565–572 (2009).
10. Laakkonen, M., Alopaeus, V. & Aittamaa, J. Validation of bubble breakage, coalescence and mass transfer models for gas-liquid dispersion in agitated vessel. *Chemical Engineering Science* **61**, 218–228 (2006).
11. Bagkeris, I., Michael, V., Prosser, R. & Kowalski, A. Modeling drop breakage using the full energy spectrum and a specific realization of turbulence anisotropy. 1–14 (2021) doi:10.1002/aic.17201.
12. Kumar, S. & Ramkrishna, D. On The Solution Of Population Balance Equations By Discretization-I. A Fixed Pivot Technique. *Chemical Engineering Science* **51**, 1311–1332 (1996).
13. Ansys® Academic Research Mechanical and CFD, Release 2020 R1.
